# Determining the Identity Nucleotides and the Energy of Binding of tRNAs to Their Aminoacyl-tRNA Synthetases Using a Simple Logistic Model

**DOI:** 10.3390/life14101328

**Published:** 2024-10-18

**Authors:** Piotr H. Pawłowski, Piotr Zielenkiewicz

**Affiliations:** 1 Institute of Biochemistry and Biophysics, Polish Academy of Sciences, 02-106 Warszawa, Poland; 2 Laboratory of Systems Biology, Institute of Experimental Plant Biology and Biotechnology, Faculty of Biology, University of Warsaw, Miecznikowa 1, 02-096 Warsaw, Poland

**Keywords:** tRNA recognition, free energy, simple logistic model

## Abstract

This study showed that the predictor in logistic regression can be applied to estimating the Gibbs free energy of tRNAs’ recognition of and binding to their aminoacyl-tRNA synthetases. Then, 24 linear logistic regression models predicting different classes of tRNAs loaded with a corresponding amino acid were trained in a machine learning classification method, reducing the misclassification error to zero. The models were based on minimal subsets of Boolean explanatory variables describing the favorite presence of nucleotides or nucleosides localized in the different parts of the tRNA. In 90% of cases, they agree with the components of the consensus strand in a class of tRNAs loaded by a given amino acid. According to the proposed theoretical model, the values of the free energy for the entry of the recognition state in the process of tRNA charging were obtained, and the inputs from identity nucleotides and the tRNA strand backbone were distinguished. Almost all the resulting models indicated leading anticodon tandems defining the first and second positions of the anticodon (positions 35 and 36 of the tRNA strand) and the small sets (up to six positions) of the other nucleotides as the natural identity nucleotides most influential in the free energy balance. The magnitude of their input to this energy depends on the position in the strand, favoring positions −1, 35, and 36. The role of position 34 is relatively smaller. These identity attributes may not always be fully arranged in a real single adaptor molecule but were comprehensively present in a given tRNA class. A detailed analysis of the resulting models showed that the absolute value of the energy of binding the tandem 35–36 decreases with the number of identity positions, as well as with the decreasing number of possible hydrogen bonds. On the other hand, in these conditions, the absolute value of the energy of binding of other identity nucleotides increases. All the models indicate that the nucleotide-independent energy of the repulsion tRNA backbone decreases with the number of identity nucleotides. It was also shown that the total free energy change in entering the recognition state increases with the amino acid mass, making this process less spontaneous, which may have an evolutionary reference.

## 1. Introduction

### 1.1. Background

Proteins perform several critical biological functions as enzymes, structural proteins, or hormones. Their synthesis occurs inside each cell in two basic stages, transcription and translation, accompanied by amino acid delivery. Transcription is a process in which a linear section of DNA encoding a protein (gene) is converted into a molecular template called messenger RNA (mRNA). During translation, the mRNA is read by ribosomes, which use the nucleotide sequence of mRNA as a recipe to synthesize a corresponding polypeptide chain of a future protein. In this process, progressing along the mRNA strand, ribosomes catalyze the formation of covalent peptide bonds between the amino acids, delivered there by specialized transfer RNA (tRNA) consisting of a few dozen nucleotides, which attach free amino acids in the cytoplasm and transport them to locations defined by the three-nucleotide sequences of mRNA (codons), which are easily recognized by the complementary three-nucleotide anticodons located at one end of a tRNA molecule. As the anticodons commonly and easily determine the amino acid load of a given tRNA, the resulting genetic code, i.e., the strict correspondence between codons and amino acids of the synthesized polypeptide, is a fundamental property of life. The basis of this unambiguous and accurate attachment of an amino acid to the CCA-3′ end of the tRNA molecule is the two-step reaction, i.e., amino acid activation with ATP and the transfer to the attachment site, catalyzed by aminoacyl-tRNA synthetases (aaRSs). The biological necessity of specific tRNA aminoacylation reactions relates to the structure of a tRNA, which determines its identity. The adequate rigidity and plasticity of typical L-shaped tRNA architecture are essential for tRNA interactions with aaRSs, requiring conformational changes and local contact of some nucleotidic residues with amino acids from a synthetase (aaRS). Thus, as suggested by crystallography, the identity is ensured by a small number of “identity nucleotides” in contact with an aaRS, predominantly located at distal regions of the tRNA molecule [1].

aaRS and tRNA recognition with identity nucleotides is the primary focus of the present study. Its innovation lies in the objective algorithmic selection of identity nucleotides and the description of the identifying interactions of tRNA and aaRSs in the language of Gibbs free energy change, i.e., the maximal work that can be completed in tRNA-aaRS binding at constant temperature and pressure. This work was estimated thanks to a proposed original theoretical model with a working hypothesis postulating the same statistical correctness of natural (thermodynamic) recognition and proper artificial classification using the machine learning algorithm of logistic regression.

### 1.2. Historical Perspective

Protein synthesis, according to the recipe written in three-letter code [2] carried by the messenger RNA (mRNA), using amino acids transported by transfer RNA (tRNA) [3] is precisely controlled by ribosomes [4]. The earlier recognition and control of a tRNA load with a given amino acid (aa) by aminoacyl-tRNA synthetases [5] is less understood. In this process, the obvious specific role of the anticodon and the universal role of the attachment site CCA-3′ may be supplemented by the other nucleotide components of the tRNA molecule (Supplementary Materials, Table S1). In the present work, we tried to confirm our expectations and find the other elements that significantly contribute to the process of tRNA loading. This intention was anticipated to be achieved by estimating their input to the change in the free energy during the recognition phase of the tRNA-loading process.

**Figure 1:**
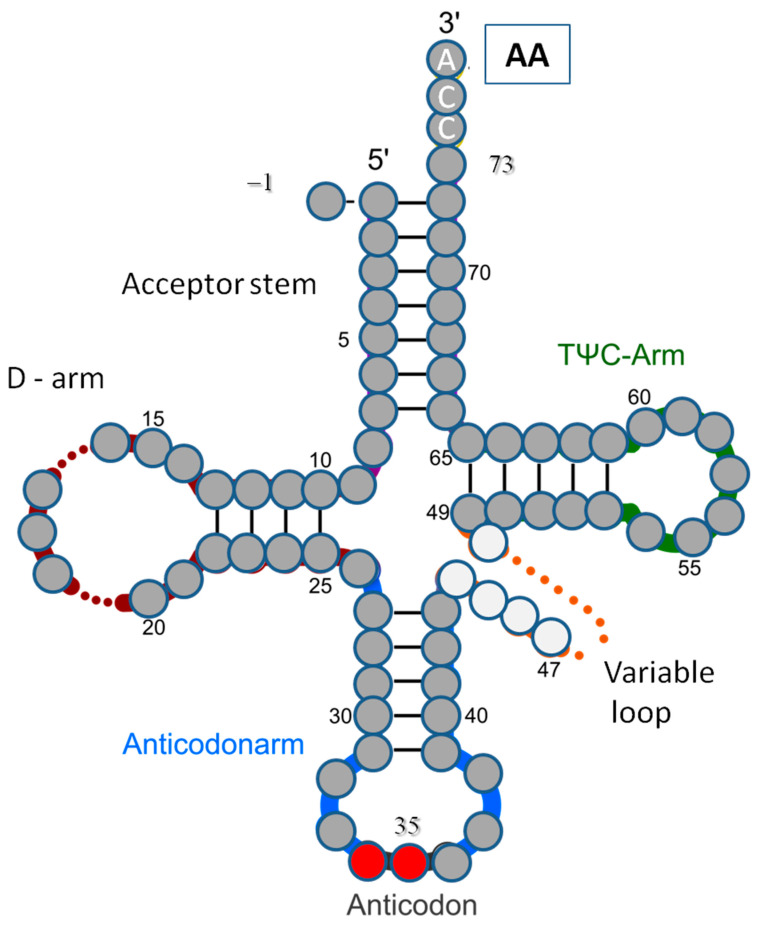
The secondary tRNA structure and the assumed numeration of positions along the strand. AA—amino acid.

The existence of supplementary recognition elements is indicated by the enzymatic “superspecifity” of synthetases. It is widely accepted that ribosomal superspecifity during translational reading is based on the complementarity of the three-letter codon and anticodon, closely exposed by mRNA and tRNA strands. This is not the case for synthetases, which, as was shown for glutaminyl-tRNA synthetase [6], may require interactions between other tRNA identity nucleotides, at very distant positions of the strand, and their protein recognition sites. These identifying elements are most commonly located in the tRNA anticodon, the acceptor stem, and the associated “discriminator” base, position 73 (Figure 1) [7]. Synthetases may sometimes recognize nucleotides in the tRNA variable pocket [8] and certain motifs of the tRNA structure [9]. The crystallographic analyses of the synthetase/tRNA complexes confirm that these macromolecules may interact in a stereochemically complementary manner [10].

The need for additional identity nucleotides may arise from the specific forward position 34 in the tRNA strand (the third place of the anticodon) and the non-complementary nature of the protein recognition site. These conditions mean that the precision of the recognition of the nucleotide occupying this place could be, in the case of tRNA charging, less than in the translational reading. Thus, to avoid pre-translational errors, these local recognitions must be supported by recognizing nucleotides in other positions. This is especially important for amino acids with a more strictly defined third letter of the code. Additionally, in the case of 6-fold degenerate amino acids, which are decoded by as many as six codons (Leu, Ser, Arg), some tRNAs for the same amino acid may expose the different leading nucleotide tandem of the anticodon, which can also cause additional uncertainty in recognition by synthetases. In this case, the long variable arm of tRNA functions as an important discrimination element [11]. Furthermore, the two forms of lysyl-tRNA synthetase [12], to operate properly, require the recognition of additional anticodon-independent identification. On the other hand, the proper fit of the tRNA acceptor site to the catalytic center of synthetase may also require additional identity nucleotides.

### 1.3. The Goal and the Idea

**Figure 2:**
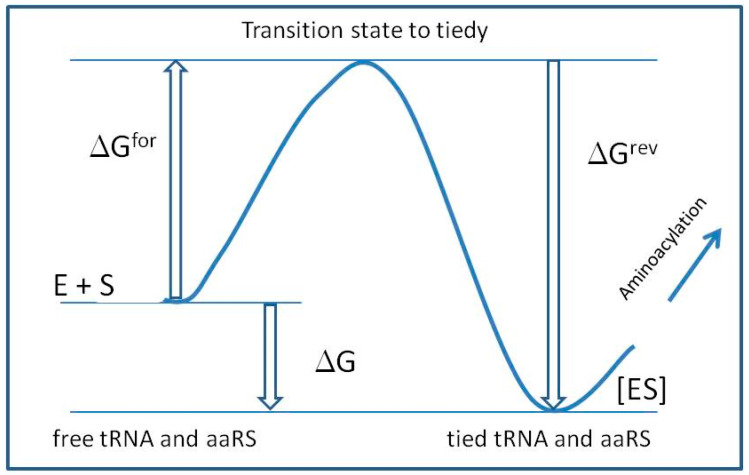
The recognition of tRNA as the thermodynamic process of crossing the energy barrier between the states of non-recognized and recognized tRNA. The symbols are as follows: S—the tRNA substrate, E—the tRNA-synthetase enzyme, [ES]—the enzyme–substrate complex (tied state), ΔG—the change in the Gibbs free energy, for—forward part, and rev—reversal part. The second (basic) transition state to aminoacetylation was signalized by an arrow.

The goal of this study is to provide an indication and a comprehensive analysis of the identity nucleotides of tRNA, crucial for its successful enzymatic recognition by aaRS. To reach this goal, the classification model explaining different amino acid loads using a selected specific nucleotide content of the tRNA strand was trained in the machine learning process, using half a thousand sequences of transfer RNA from database data for 90 different species. The idea came from the working hypothesis that natural recognition and proper artificial classification both involve the extraction and analysis of the object attributes, namely, identity nucleotides, leading to semantically the same result. Thus, a proper classification method could offer a theoretical model of natural recognition of tRNA by the aminoacyl-tRNA synthetases and reveal the identity molecules, their type, and position. As a consequence, we may expect that the ratio of the probability of classification success to the probability of classification error equals the odds of the recognition process in nature. Assuming that the natural recognition resembles the thermodynamic process of crossing the energy barrier between the states of non-recognized and recognized tRNA (in both directions), we could estimate the energetic constraints of this process. In this picture (Figure 2), the energy to be overcome (ΔG^for^) represents the energy required to bring the tRNA closer and place it in the correct distance and orientation relative to the synthetase anticodon binding domain and the catalytic center. The decrease in the energy after crossing the energy barrier (ΔG^rev^) is a measure of the stability of the tRNA-synthetase complex achieved through the local interactions necessary for the catalysis of esterification of an amino acid to the 3′ end of a tRNA. In the discussed case, ΔG = ΔG^for^ + ΔG^rev^, where ΔG^for^ > 0 and ΔG^rev^ < 0; thus, ΔG^for^ corresponds to the repulsion, and ΔG^rev^ corresponds to the attraction between the tRNA and its aminoacyl-tRNA synthetase.

According to the presented picture, the free and tied states of tRNA and aaRS are separated by the transition state to tidy, in which intermolecular attraction and repulsion balance each other. This transition is maximally repulsive for a longer distance and partially stabilized for a shorter distance before the next step in the process of tRNA loading with an amino acid. We may expect that ΔG^for^ is built by effectively repulsing the long-range interactions (e.g., electrostatics and hydrophobic) and ΔG^rev^ by effectively attracting the short-range interactions of the selected tRNA nucleotides and aaRS amino acids. In this scheme, certain nucleotides may have the opposite, repulsive effect but not change the overall picture of the short-range attraction.

It is worth adding that artificial classification can be performed using different tools, such as simple observations and basic statistical analysis. This paper proposes a machine learning algorithm offering fast big data analysis and objectivity of algorithmic decisions. The advantage of this method is the objective automatic selection of identity nucleotides and analytical description of the recognition process using the so-called predictor function, which quantitatively scores the contribution of each identity nucleotide to the selection of a given tRNA class (amino acid load) and, in this way, evaluates its importance. An inherent positive feature of the machine learning method is that it automatically improves the classification quality through experience.

### 1.4. The Importance of Positions—Initial Findings

To perform classification tasks, 24 different classes of tRNA were considered. They were distinguished by the charging amino acids and, if applied, by the different forms of synthetase (i.e., LysI and LysII) [13] or the specific degeneration in the first position of the anticodon (e.g., Leu1 = {GA *} and Leu2 = {AA *}). As attributes of the classes, nucleotides or nucleosides at a given position in the tRNA strand were used (−1, 1, 2, 3…76).

**Figure 3:**
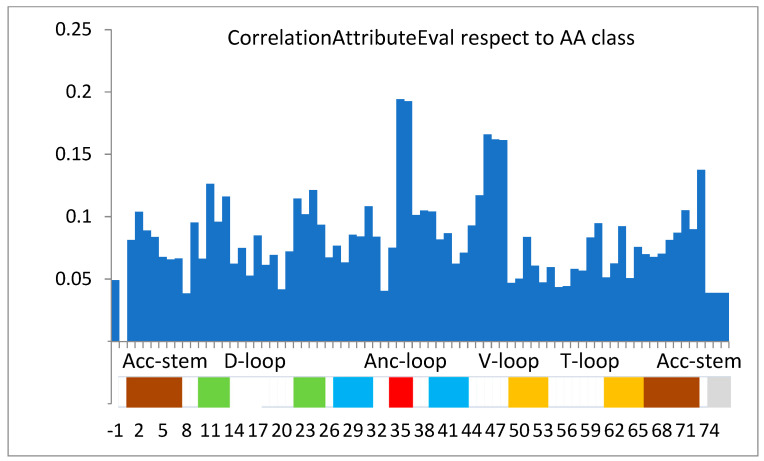
*The attribute importance ranking evaluated for the full training set using CorrelationAttributeEval. The correlation of the attributes with the amino acid class was analyzed.* The colored area at the bottom indicates parts of the secondary structure of the tRNA molecule. The white area indicates loops, the red area indicates anticodons, and the same colors indicate complementary regions of stems. Position 34 (the 3rd letter of the anticodon) is surprisingly less important than other non-coding positions.

One may expect the dominant role of the anticodon in determining the amino acid content of tRNA. The preliminary attempts with the correlation attribute evaluator (Figure 3) showed the highest importance of positions 35 and 36 of the tRNA strand and, surprisingly, relatively low importance of the 3rd letter in the anticodon (positions 34 of tRNA), which is in 41st place. The latter finding confirms the need to look for the other identity nucleotides and, thus, in light of the previous comments, for a theoretical classifier properly modeling natural recognition, which is the topic of the presented work. Thus, the central research question is “Do the nucleotides beyond the anticodon in tRNA significantly contribute to the specificity of aaRS recognition and loading of amino acids?”

### 1.5. General Observations

**Figure 4:**
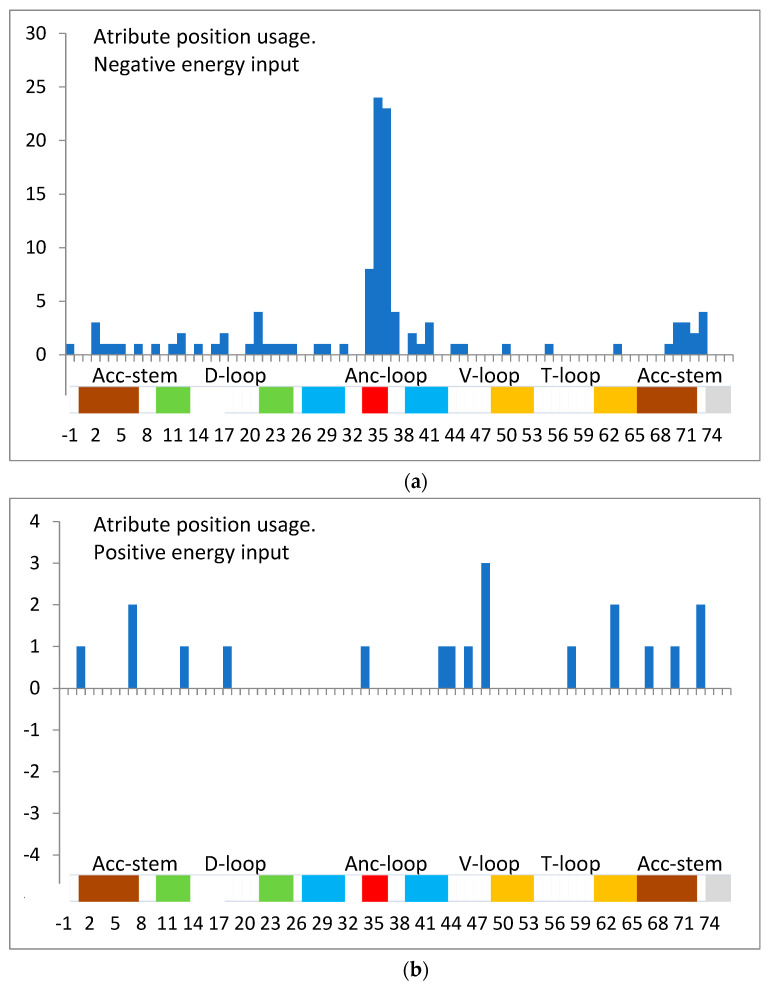
(**a**). A simple logistic model of tRNA recognition. The usage of positions with a negative input (attraction) to change the free energy. (**b**). A simple logistic model of tRNA recognition. The usage of positions with positive (repulsion) input to change the free energy. The white area indicates loops, the red area indicates anticodons, and the same colors indicate complementary regions of stems.

**Figure 5:**
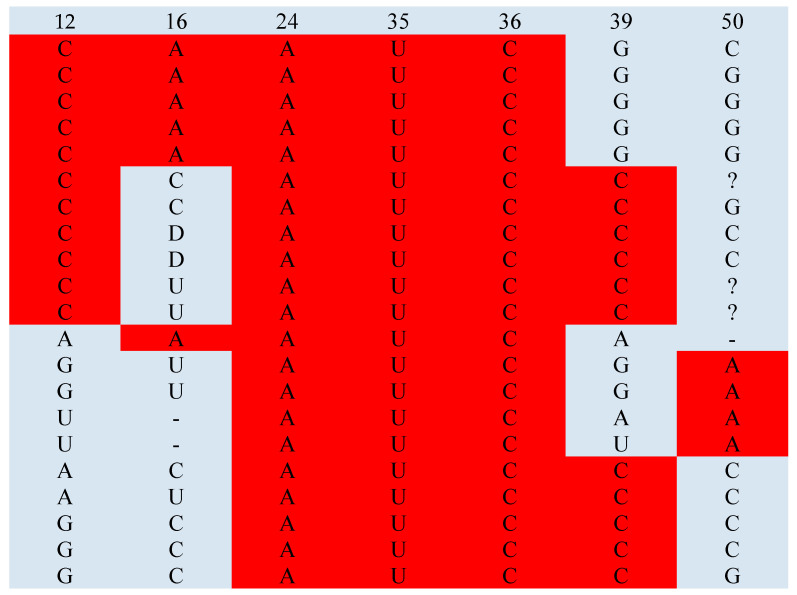
An example of identity nucleotides (red) found in the seven positions, i.e., 12, 16, 24, 35, 36, 39, and 50, in the analyzed 21 real tRNA^Glu^ strands (for glutamine) using the simple logistic model. Other non-identity nucleotides in these positions (gray) are also shown. In this case, the mean coverage by the identity nucleotides is 64.6% of the possible area. There is no full representation of identity nucleotides in one strand. The figure illustrates the scale of the considered phenomenon, which is also common in other classes. The symbol “?” is 5-methylcytidine.

A proper classifier for the best assignment of amino acid to a given tRNA was revealed in preliminary numeric experiments. It builds linear logistic regression models, minimizing logistic loss with the LogitBoost algorithm [14,15]. With this classifier, 100% accurate models for amino acid load were obtained, describing the significant positive or negative impact of choosing a given nucleotide at a given position. This way, the most important ensembles (up to eight positions) and their members in the tRNA strand (adding negative or positive input to the free energy) could be indicated, especially in the tRNA anticodon and the position 37, and also in the acceptor stem at the 5′ end position 2, at the 3′ end position 73, in the D-loop position 21 (Figure 4a) and in the variable loop position 48 (Figure 4b). Statistical analysis of the real tRNA sequences showed that the average of the minimal occurrence of the representants of possible identity nucleotides in a given class is 71 ± 15%. This means they may not always be fully arranged in a real single adaptor molecule but were comprehensively present in a given tRNA class (Figure 5). The full occurrence of predicted identity nucleotides, at least in one strand, was only observed in the case of 67% of tRNA classes.

### 1.6. Main Findings

In the detailed analysis and according to the working hypothesis, when crossing the energy barrier, the values of free energies—forward and reverse, entering and returning from the recognition state in the process of tRNA charging—were obtained, as well as contributions to the Gibbs free energy change. The latter was also possible due to the specific advantage of logistic regression model prediction functions, which, with the assumed correctness of the working hypothesis, may be easily interpreted as non-dimensional changes in the free energy, which was used in the proposed theoretical model (see the Materials and Methods). The magnitude of the identity nucleotide input to the discussed energy depends on the position in the strand, favoring positions −1, 35, and 36. Detailed analysis of the resulting models shows that the height of the reverse barrier related to anticodon tandems decreases with the number of the identity nucleotides, corresponding to the decreasing hydrogen bonding. On the other hand, the height of the reverse barrier, related to the other identity nucleotides, increases with the total number of indication nucleotides. Similarly, the total free energy change in entering the binding state increases with the amino acid mass (see the Discussion). It appears that, apart from the anticodon, the identity nucleotides add additional binding energy to overcome a certain energy level specific to a given amino acid class. Thus, the universal genetic code is supported by a precisely distributed quantity of binding energy in the tRNA–ligase interaction. However, this process is not efficient enough to keep the strong binding unchanged, such as for tRNAs transporting low-mass amino acids. It was also noticed that some subsets of the identity nucleotides, together with the anticodon tandem, are always and only present in a given tRNA class, being universal markers. Some other subsets, even without anticodon tandem, are unique, i.e., they may only be present in the tRNA of a given class, but they are not universal. Both define something like a pre-translational recognition code. The above findings indicate the possible mechanical and informational role of the unique sites outside the anticodon and raise questions about the direction of the evolution of these sites, which we attempt to answer in the Discussion.

## 2. Materials and Methods

### 2.1. Data Collection

Data containing tRNA sequences (nucleic, mitochondrion, and plastids) of molecules transferring complementary amino acid (aa) were taken from the tRNAdb—Transfer RNA Database [16] (link: http://trnadb.bioinf.uni-leipzig.de accessed on 28 March 2023). The data were reviewed to prevent duplicates, and the issues containing non-standard bases (A, U, G, and C) in positions 35 and 36 were excluded. Finally, 511 issues were collected as a training set of attributes describing nucleotides or nucleosides at positions −1, 1, 2, 3…76, and the class attribute. They are found in three kingdoms, i.e., Bacteria (140), Archaea (68), and Eukaryota (303), represented by 90 different organisms. The assumed position numeration is presented in Figure 1. The list of the 67 nucleot(s)ides considered and their derivatives, used symbols, and one-letter codes is presented in Supplementary Materials, Table S1. The empty positions were also considered.

The 24 different classes of tRNA were considered. They were defined according to the charging amino acids. In the case of a 2-fold degenerate first and/or second anticodon position (Arg, Leu, Ser), an additional class was attributed (e.g., Ser = {AG *}, Ser2 = {UC *}). The tRNA loaded using different forms of lysyl-tRNA synthetase (i.e., LysI and LysII) were also distinguished. A list of distinguished classes, tRNA^aa^, is presented in Table 1.

In the data analysis, the collection of machine learning algorithms for data mining tasks was performed with Weka 3.8.5 [17].

### 2.2. Preliminary Experiments Methods

In preliminary experiments, the attribute importance (Pearson’s correlation ranking between the attribute and the class) for the full training set was evaluated using CorrelationAttributeEval, with the Ranker -T -1.7976931348623157E308 -N -1.

To find the classifier algorithm of the best predictability of the tRNA class, five different classifiers were trained on a full training set: Dl4jMlpClassifier, LibLINEAR, RandomForest, SimpleLogistic, and SMO. The benchmark was determined using the ZeroR algorithm. Parameters of the training processes were accepted as pre-defined in the Weka environment, except the SimpleLogistic classifier, where an option of useCrossValidation = False was chosen to prevent the data order influence. The classifiers were evaluated using the correctness of predictions in a 66% split, 10-fold cross-validation, and full training set.

### 2.3. Final Experiments Methods

Finally, the classification tasks with the chosen SimpleLogistic classifier, minimizing misclassification error for the training set, were performed using the *weka.classifiers.functions* scheme: *SimpleLogistic -I 0 -S -M 500 -H 50 -W 0.0*. Linear predictor function f_i_ for amino acid class aa_i_ was used in the following form:

f_i_: = b_i_ + Σ_jk_ p_ijk_ × [pos_ij_ = N_k_]  i = 1…24, j = −1…76, k = 1…67 (1)

where [pos_ij_ = N_k_] is a Boolean explanatory variable (value of 0 or 1) for the tRNA from class AA_i_, describing the occurrence at the strand position j, the nucleot(s)ide N_k_. In linear regression, p_ijk_ is the parameter indicating the relative effect of a particular explanatory variable on the value of the predictor, and b_i_ is the bias, describing the part of predictor value that cannot be explained by the specific criterion postulated by the model. The symbol Σ_jk_ indicates the sum of components indicated by indexes j and k. In the considered case, index i corresponds to Table 1, strand position j corresponds to Figure 1, and nucleotide k corresponds to Supplementary Materials, Table S1.

### 2.4. Statistical Analysis

A detailed revision of the examined real tRNA strands and statistical analysis of the results of the SimpleLogistic classification were performed to obtain consensus strains, the histograms of position usage, and the averages of considered energies using standard formulas in the MO 2007 Excel calculation sheet (Microsoft Corporation, Redmond, WA, USA, 2018, link: https://office.microsoft.com).

### 2.5. The Theoretical Model of Machine Learning Simulation of tRNA Binding to Aminoacyl-tRNA Synthetase

Let us consider the intermediate stage of tRNA charging [18], i.e., the recognized tRNA^aa^ binding to the corresponding preloaded [Aminoacyl-AMP aaRS] complex

tRNA^aa^ + [Aminoacyl-AMP aaRS] → [tRNA^aa^ Aminoacyl-AMP aaRS] (2)

for each amino acid (aa) and aminoacyl-tRNA synthetase (aaRS) of the genetic code. The 24 amino acid classes were considered. This number led to distinguishing binding of the tRNA for the same amino acid but with different anticodons, positions 35 and 36, and tandems, as for Leu, Arg, and Ser. Furthermore, two classes of lysyl-trna synthetases may be considered.

When [Aminoacyl-AMP aaRS] complexes for all coded amino acids are equally available, the probability p_ii_ of binding the tRNA^aa^_i_ for the i-th amino acid to the corresponding *i*-th complex may be described by the Boltzmann factors as

(3) $$p_{ii}=Exp(-\frac{{\Delta G}_{ii}}{k_{B}T})/(1+\sum_{j=1}^{n}Exp(-\frac{{\Delta G}_{ij}}{k_{B}T}))\;\;i=1,\ldots 24$$

where ΔG_ij_ is the Gibbs free energy of binding the tRNA^aa^_i_ to the j-th preloaded complex (for the *j*-th amino acid), T is the absolute temperature [K], k_B_ is Boltzmann’s constant, and n is the number of coded amino acids.

If it is assumed that properly recognized tRNA^aa^_i_ binds much stronger than others (ΔG_ii_ ▯ ΔG_ij_), the above distribution (Equation (3)) can be simplified as

(4) $$p_{ii}=Exp(-\frac{{\Delta G}_{ii}}{k_{B}T})/(1+Exp(-\frac{{\Delta G}_{ii}}{k_{B}T}))\;\;i=1,\ldots 24$$

Thus, the logit function, or ln(odds_i_), defined for different i

logit(p_ii_) = ln(p_ii_/(1 − p_ii_))  i = 1, … 24 (5)

can be expressed as

logit(p_ii_) = −ΔG_ii_/k_B_T  i = 1, … 24 (6)

The above rewritten equation allows the assignment of probabilistic meaning to the free energy of binding, i.e.,

ΔG_ii_ = − k_B_T logit(p_ii_)  i = 1, … 24 (7)

If we assume that the same thermodynamic conditions were maintained during the whole evolution of the universal genetic code and the probability of evolutional classifying of a given tRNA to a certain tRNA aa class equals the thermodynamic probability of the tRNA binding to the preload complex of the corresponding synthetase, then the logit(p_ii_), and thus ΔG_ii_, may be considered the physical determinants of the tRNA classes during the evolution.

Furthermore, if we assume that ΔG_ij_ for a given synthetase depends on the varying contents of the tRNA nucleotide sequence and the evolutional selection with logit(p_ii_) as a determinant resembles the classification algorithm with the logistic models ln(odds’_i_) = f_i_, where f_i_ is the predictor function for a given class aa_i_ predicting the tRNA class from its sequence, then the theoretical classification algorithm can model the evolutional selection, and the ln(odds’_i_) may approximate logit(p_ii_).

Finally, we can put

logit(p_ii_) = f_i_  i = 1, … 24 (8)

Then, combining Equations (7) and (8) results in

ΔG_ii_ = −k_B_Tf_i_  i = 1, … 24 (9)

which shows that f_i_ may be considered a dimensionless analog of the free energy of binding.

Consequently, for f_i_ described by Equation (1), the single energy inputs from the *j*-th position of the tRNA strand, occupied by the k-th nucleot(s)ide, equals

ΔG_ijk_ = −k_B_Tp_ijk_ × [pos_ij_ = N_k_]  i = 1, … 24, j = −1…76, k = 1…67 (10)

Then, the part of the energy, position, and nucleotide independent may be calculated as

ΔG^0^_ii_ = −k_B_Tb_i_  i = 1, … 24, (11)

When dividing the total free energy into forward and reverse parts (Figure 2), i.e.,

ΔG_ii_ = ΔG^for^_ii_ + ΔG^rev^_ii_  i = 1, … 24, (12)

for the long-range repulsion, it is reasonable to approximate the ΔG^for^_ii_ using the nucleotide-independent part of the binding energy change, i.e.,

ΔG^for^_ii_ = ΔG^0^_ii_  i = 1, … 24, (13)

and ΔG^rev^_ii_ via the sum (∑_jk_) of nucleotide-dependent inputs, i.e.,

ΔG^rev^_ii_ = ∑_jk_ ΔG_ijk_.  i = 1, … 24, (14)

with j and k, as defined in Equation (1).

Expected in this approximation, ΔG^for^_ii_ > 0 estimates the repulsion energy at the tRNA tRNA-synthetase approaching, and ΔG^rev^_ii_ < 0 estimates the attraction energy of the arising bonds at the return.

## 3. Results

### 3.1. Preliminary Experiments

The attribute-importance ranking evaluated for the full training set using CorrelationAttributeEval is presented in Figure 3. The correlation rank values obtained for dominating attributes are presented in Table 2. It is worth emphasizing that the third position of the anticodon only takes the 41 position in this rank.

The results of efforts toward finding the classifier algorithm of the best predictability of the tRNA class are presented in Table 3. According to the above findings, the SimpleLogistic classifier was chosen for the following machine learning experiments. The advantage of this algorithm is the automatic selection of the most important attributes, being natural candidates for the identity nucleotides.

### 3.2. Final Classification Task

**Figure 6:**
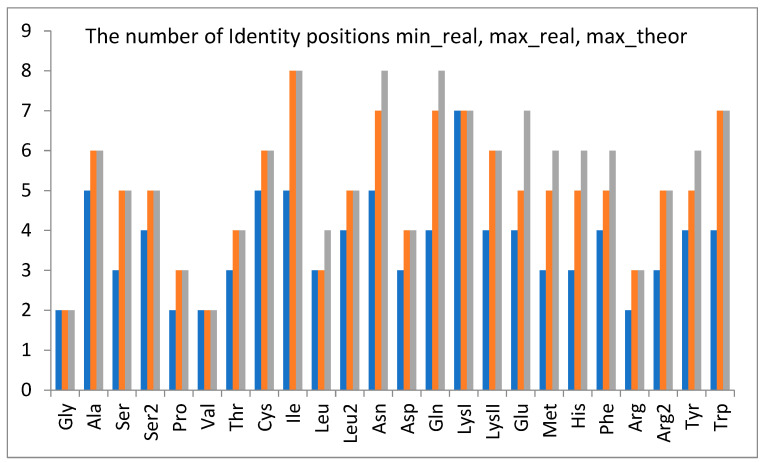
The number of identity positions, real and possible cases, found with the SimpleLogistic model for different tRNA^aa^ classes. Color meaning: blue—min_real, the minimal number found in the real tRNA^aa^ strands; orange—max_real, the maximal number found in the real tRNA^aa^ strands; grey—max_theor, the maximal number theoretically predicted by the SimpleLogistic model for the strand of a given tRNA^aa^ class. The tRNA^aa^ classes are presented according to increasing amino acid molecular weight.

**Figure 7:**
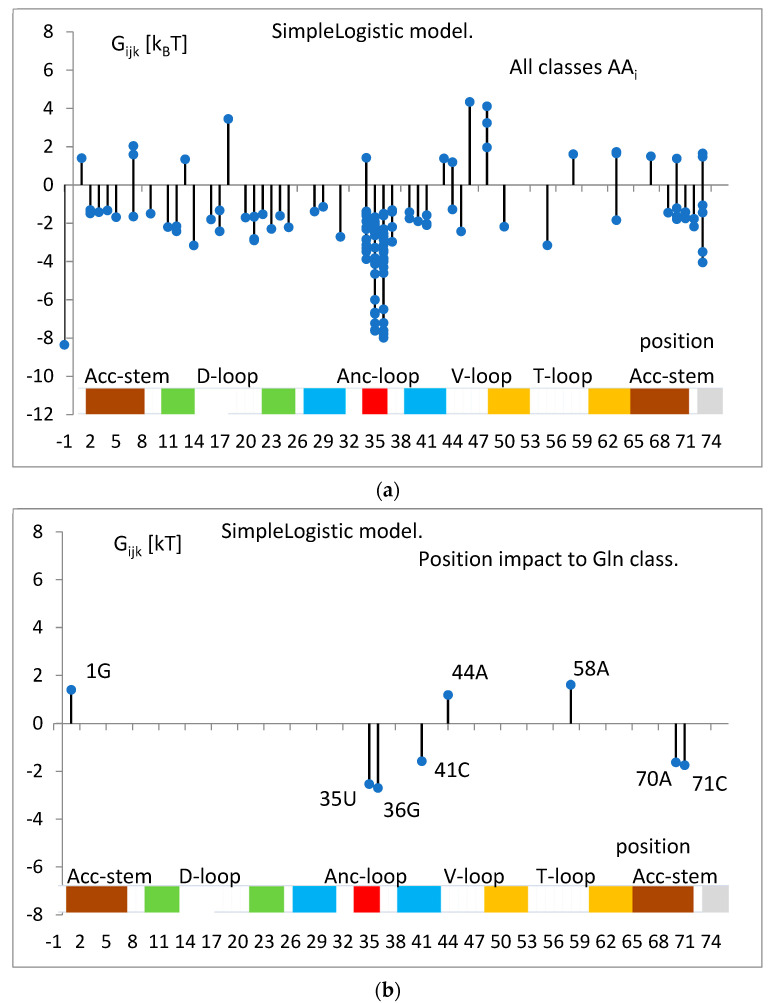
(**a**). The landscape of all inputs to the free energy, ΔG_ijk_, determined in the SimpleLogistic model for the full training set. ΔG_ijk_ is the input to the free energy of tRNA binding to the *i*-th synthetase from the *j*-th position of the tRNA strand occupied by the k-th nucleot(s)ide. All classes and all nucleot (s)ides are considered. The point represents a single energy input value, positive or negative. Vertical bars show the maximal or minimal value. The colored area at the bottom indicates parts of the secondary structure of the tRNA molecule. The white area indicates loops, the red area indicates anticodons, and the other same colors indicate complementary regions of stems. (**b**). The example for the tRNA^Gln^ class is extracted from (**a**).

**Figure 8:**
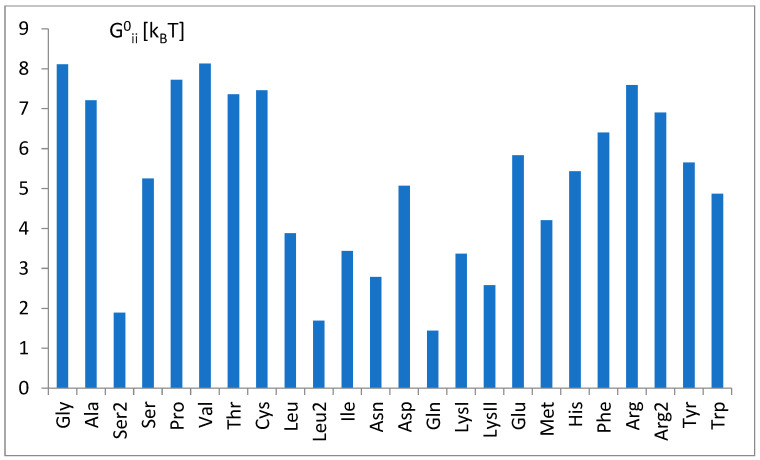
The nucleotide-independent part of the free energy change, ΔG^0^_ii_, for different tRNA^aa^ classes, calculated according to parameters of the SimpleLogistic classification, b_i_ (Equation (1)), and the proposed formula of the theoretical model of tRNA tRNA-synthetase binding (Equation (11)). The tRNA^aa^ classes are presented according to increasing amino acid molecular weight.

**Figure 9:**
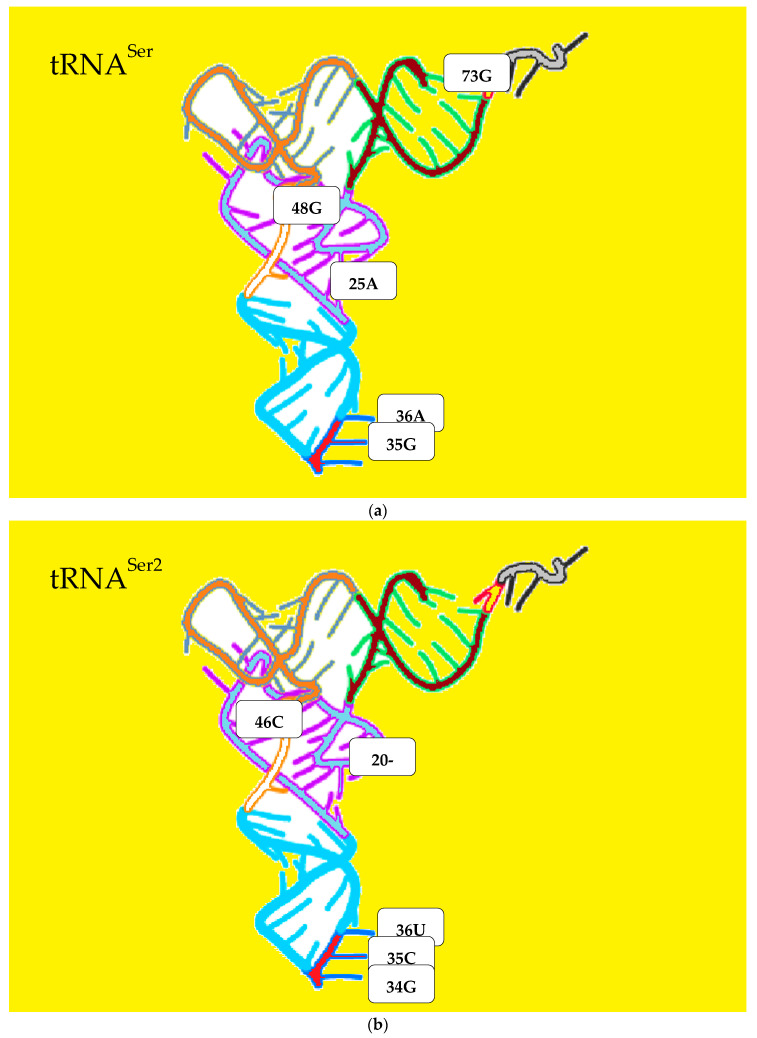
(**a**). The spatial distribution of identity nucleotides for class tRNA^Ser^. (**b**). The spatial distribution of identity nucleotides for class tRNA^Ser2^. Colors corresponds to those in Figure 3, Figure 4a,b, and Figure 7a,b.

To find the identity nucleot(s)ides, classification with the SimpleLogistic classifier was performed with the full training set. The final parameters are presented in Supplementary Materials, Table S2. The resulting model selects the most important positions and their nucleotidic contents, i.e., the nucleot(s)ides with a significant, positive, or negative (~) impact on a value of predictor function (Equation (1)), and the value of a change in the free energy of binding (Equations (9)–(11)). The revealed findings are presented in Figure 4, Figure 5, Figure 6, Figure 7, Figure 8 and Figure 9, Table 4 and Table 5.

The histograms of attribute position usage, separately for positive and negative inputs, are summarized in Figure 4a and Figure 4b, respectively. The negative-input positions 2, 21, 34, 35, 36, 37, and 73 and positive-input position 48 are dominant. They represent anticodons with the neighborhood, acceptor stem, D-loop, and V-loop. Here, in sum, the possible alternative filling in position 34 (degeneration) is neglected.

An example of an individual tRNA class is shown in Figure 5, illustrating that attributes may not always be fully arranged in a real single adaptor molecule but are comprehensively present in a given tRNA^aa^ class. The full sets (max_theor) of predicted class identity nucleotides that occurred at least in one strand were observed in 67% (16/24) of tRNA classes (Figure 6). In other classes, the maximum occurrence (max_real) is at least 71% (5/7) for strands. The minimal minimum (min_real) is 50%, and its average value in all classes is 71 ± 15%.

The landscape of determined Gibbs free energy inputs ΔG_ijk_ calculated with the parameters p_ijk_ of the trained SimpleLogistic model (Equation (1)) and the theoretical thermodynamical model of binding, assigning to the identity nucleotides of a given tRNA^aa^ class the free energy inputs (Equation (10)), is presented in Figure 7a. The domination of anticodon tandem and positions −1 and 73 is visible. The extracted example for a single class, tRNA^Gln^, is shown in Figure 7b.

The nucleotide-independent part of free energy change, calculated according to parameters b_i_ (Equation (1)) and the theoretical formula (Equation (11)), is presented in Figure 8.

The possible identity nucleotides selected for different tRNA^aa^ classes using the SimpleLogistic classifier are presented in Table 4. The symbol “~” indicates nucleotides of positive energy input (repulsion) (for other symbol meanings please see Supplementary Materials, Table S1). Some cases in position 34 contain more than one nucleot(s)ide (degeneration). The maximum number of possible identity positions per class is eight. The minimum number is two. Position 34 (the third position of the anticodon) is present in 33% (8/24) of classes. Classes with the same anticodon tandems (e.g., Asp and Glu, Gln and His, and Lys I and LysII) indicate different sets of the other identity nucleotides.

The predicted identity positions were compared with those from consensus representation. In Table 5, the consensus representatives (columns) consistent (red) and inconsistent (gray) with predictions of the SimpleLogistic model are indicated. The elements differing from the attributes of negative impact were treated as consistent. A total of 90% (116/129) of predicted identity nucleotides of non-positive input to energy (non-repulsing) agree with the nucleotides in consensus strands for respective tRNA^aa^ classes.

An example of the spatial distribution of identity nucleotides for twin classes tRNA^Ser^ and tRNA^Ser2^ is presented in Figure 9a,b. This example shows that single Seryl-tRNA synthetase can recognize two ensembles of identity nucleotides.

### 3.3. The Free Energy Considerations

**Figure 10:**
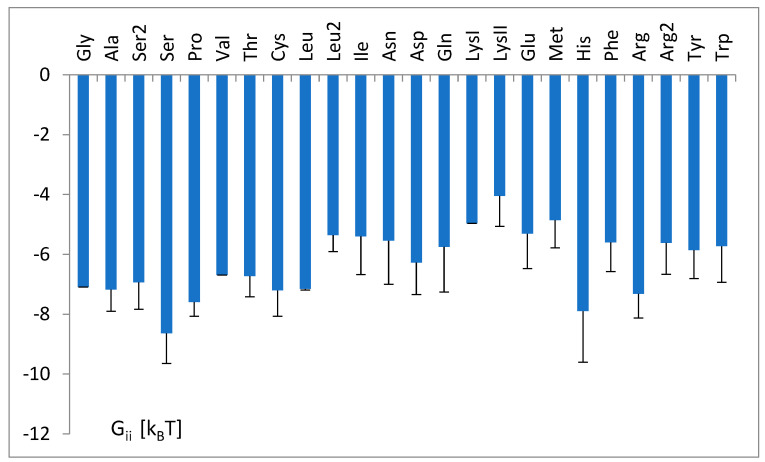
The average values of the change in the free energy of binding ΔG_ii_ for each tRNA^aa^ class determined using Equation (9) and the parameters of Equation (1) taken from the classification task with the SimpleLogistic classifier. The standard deviation is included. The tRNA^aa^ classes are presented according to increasing amino acid molecular weight.

The change in free energy of binding the tRNA^aa^ to aa-tRNA synthetase was calculated for each tRNA strand in the training set according to Equation (9) and the parameters of Equation (1). This was determined using the SimpleLogistic classifier in the final classification task modeling the ln(odss) of the recognition of tRNA with a given set of identity nucleotides via the linear combination of the Boolean variables characterizing the occurrence of nucleotides and the non-specific bias. Then, the average values of ΔG_ii_ for each tRNA^aa^ class were determined (Figure 10). The most negative ΔG_ii_ shows the tRNA^Ser^ class: ΔG_ii_ = −8.6 [k_B_T]. The least “negative” is the tRNA^LysII^ class: ΔG_ii_ = −4.0 [k_B_T].

**Figure 11:**
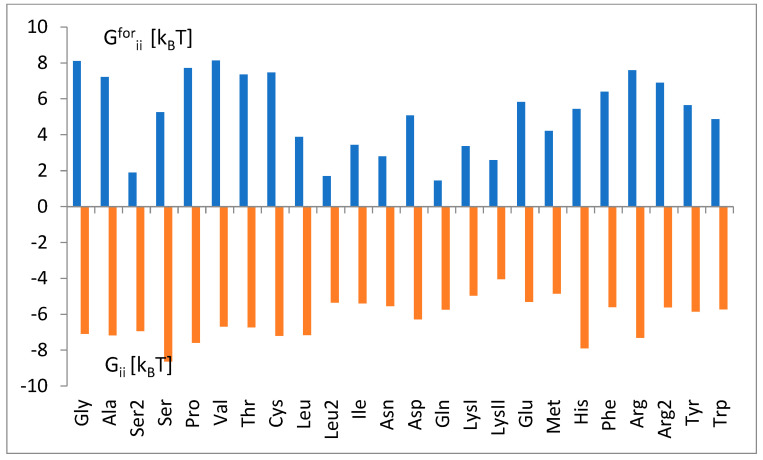
The values of ΔG_ii_ and ΔG^for^_ii_ (=ΔG^0^_ii_) are summarized on one chart to estimate strong ΔG^rev^_ii_ = ΔG_ii_ − ΔG^for^_ii_ as a measure of the strength of tRNA and aminoacyl-tRNA synthetase attraction. The values are taken from Figure 8 and Figure 10. The tRNA^aa^ classes are presented according to increasing amino acid molecular weight.

To approximately illustrate the strength of tRNA and aminoacyl-tRNA synthetase attraction, ΔG_ii_ and ΔG^for^_ii_ (=ΔG^0^_ii_) were summarized on one chart (Figure 11). The strongest attraction, ΔG^rev^_ii_ = ΔG_ii_ − ΔG^for^_ii_ (Equations (12) and (13)), was observed for the tRNA^Gly^ class, ΔG^rev^_ii_ = −15.2 [k_B_T]. The weakness attraction, ΔG^rev^_ii_ = −6.6 [k_B_T], was observed for the tRNA^LysII^ class.

**Figure 12:**
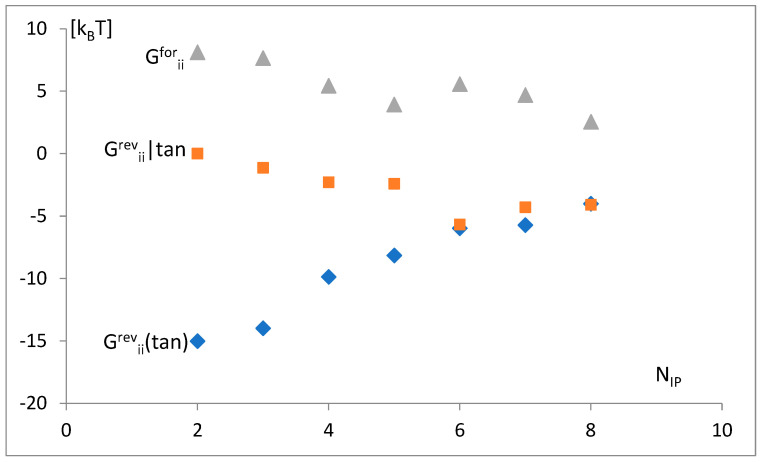
The simultaneous dependence of free energy parts—reversal limited only to the anticodon tandem, ΔG^rev^_ii_(tan); the rest of the reversal energy without the anticodon tandem, ΔG^rev^_ii_|tan; and forward, ΔG^for^_ii_, on the maximal number of possible identity points, N_IP_. Standard variations are 1.45, 1.49, and 1.21, respectively.

The simultaneous dependence of free energy parts—forward ΔG^for^_ii_, the reversal limited only to the anticodon tandem ΔG^rev^_ii_(tan), and the rest of the reversal energy without the anticodon tandem ΔG^rev^_ii_|tan—on the maximal number of possible identity points N_IP_ is shown in (Figure 12). A decrease is shown in the attraction of the anticodon tandem, ΔG^rev^_ii_(tan); a simultaneous decrease in non-specific repulsion, ΔG^for^_ii_; and an increase in attraction of other identity nucleotides, ΔG^rev^_ii_|tan.

**Figure 13:**
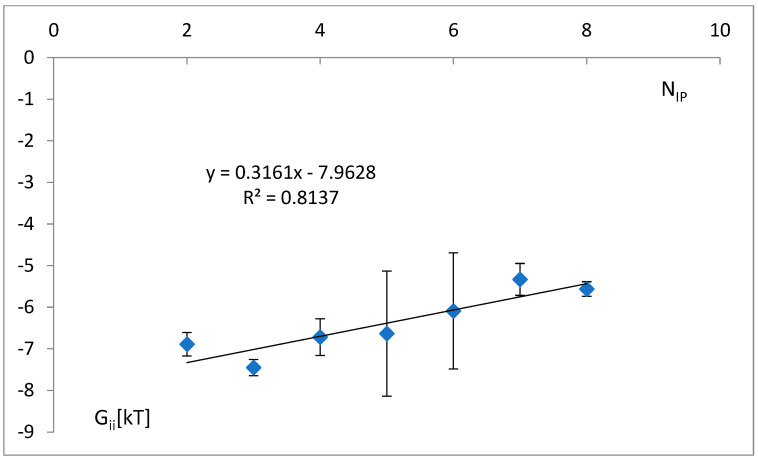
The dependence of the change in total free energy ΔG_ii_ on N_IP_. Standard variations are shown.

The decrease in the attraction of anticodon is not sufficiently balanced by an increase in the attraction of other identity points and a decrease in non-specific repulsion. Thus, the total free energy change increases with N_IP_ (Figure 13).

### 3.4. The Analysis of the Attraction of Anticodon Tandem

**Figure 14:**
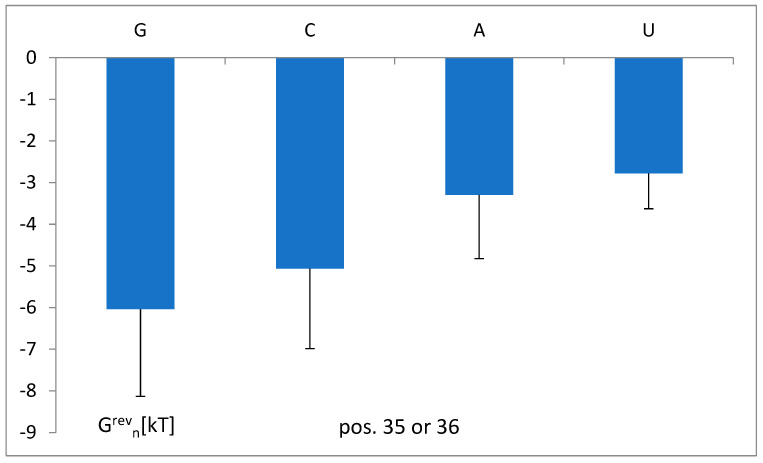
The dependence of the energy of tRNA attraction to aminoacyl-tRNA synthetase in the area of the anticodon tandem on the anticodon contents (G, C, A, and U). ΔG^rev^_n_ is the reversal energy limited to only one position (35 or 36) and one nucleotide type. Standard variations are shown.

The dependence of the energy of tRNA attraction to aminoacyl-tRNA synthetase in the area of the anticodon tandem on the nucleotide contents is shown in Figure 14. The measure of this attraction ΔG^rev^_n_, and the average reversal energy limited only to the one position (35 or 36) and the one nucleotide type, increases for “weak” nucleotides (A and U), with possibly less hydrogen bonding. The 24 classes were considered.

**Figure 15:**
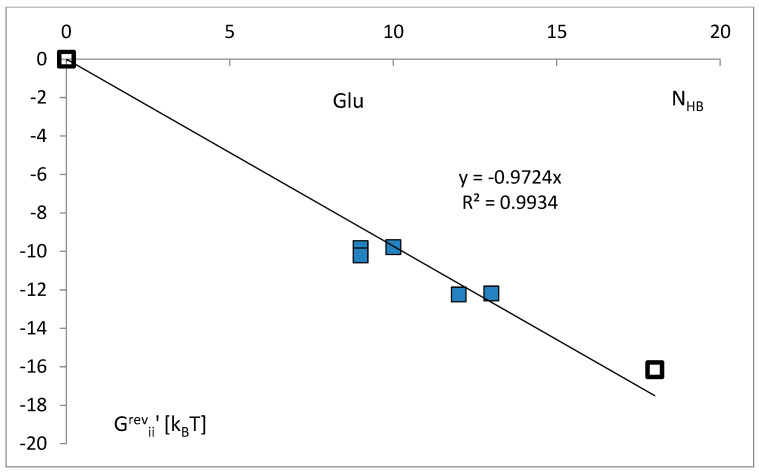
The dependence of the total energy of the attraction on the actual number of possible hydrogen bonds in a given real identity ensemble of the tRNA^Glu^ class. ΔG^rev^_ii’_ represents the reversal part of free energy change for real cases of the same subsets of identity nucleotides in the tRNA^Glu^ strands with the anticodon tandem 35U and 36C. Empty squares represent a priori zero and the theoretical value of the free energy change for the maximal number of hydrogen bonds in the case of all identity nucleotides presented.

The example of the dependence of the total energy of the attraction on the actual number of possible hydrogen bonds in a different identity ensemble of the tRNA^Glu^ class (Figure 15) confirms the above findings. At the constant anticodon tandem, the attraction of the other identity nucleotides increases, so actual ΔG^rev^_ii’_ decreases with the number of hydrogen bonds. Here, ΔG^rev^_ii’_ was not averaged over all classes but represents real cases of the same subsets of identity nucleotides.

### 3.5. The Free Energy and Recognition

**Figure 16:**
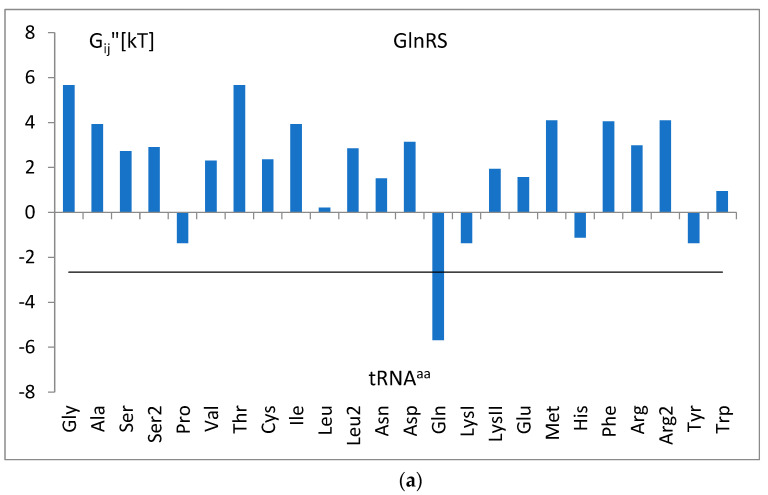

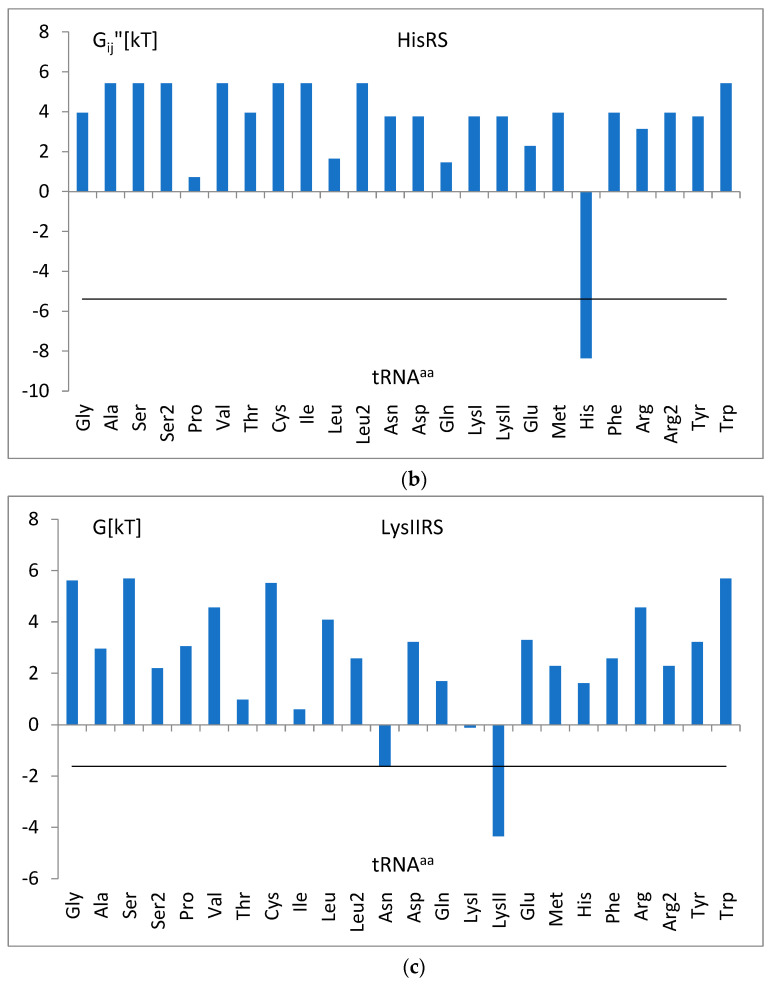
(**a**). Consensus free energy change ΔG_ij_” estimated with the parameters of the final classification task but for the nucleotides present in the consensus strands of different classes. The horizontal line represents the energy of the reduced binding real tRNA^Gln^ strand. The tRNA^aa^ classes are presented according to increasing amino acid molecular weight. (**b**). The consensus free energy change ΔG_ij_” estimated with the parameters of the final classification task but for the nucleotides present in the consensus strands of different classes. The horizontal line represents the energy of the reduced binding real tRNA^His^ strands. The tRNA^aa^ classes are presented according to increasing amino acid molecular weight. (**c**). The consensus free energy change ΔG_ij_” estimated with the parameters of the final classification task but for the nucleotides present in the consensus strands of different classes. The horizontal line represents the energy of the reduced binding real tRNA^LysII^ strands. The tRNA^aa^ classes are presented according to increasing amino acid molecular weight.

To analyze the interaction of tRNAs of different classes (i) with the given aminoacyl-tRNA synthetase (j), Equations (1) and (9) with the parameters of the final classification task were applied to the nucleotides of the tRNA consensus strands (Table 5). This enabled estimating the consensus free energy change ΔG_ij_”. The examples for glutaminyl-tRNA synthetase (Figure 16a), histidyl-tRNA synthetase (Figure 16b), and lysylI-tRNA synthetase (Figure 16c) are shown. The results suggest that to be properly recognized, the change in binding free energy should decrease below a certain level, characteristic for a given class. The weaker bound or repelled tRNAs are not recognized.

**Figure 17:**
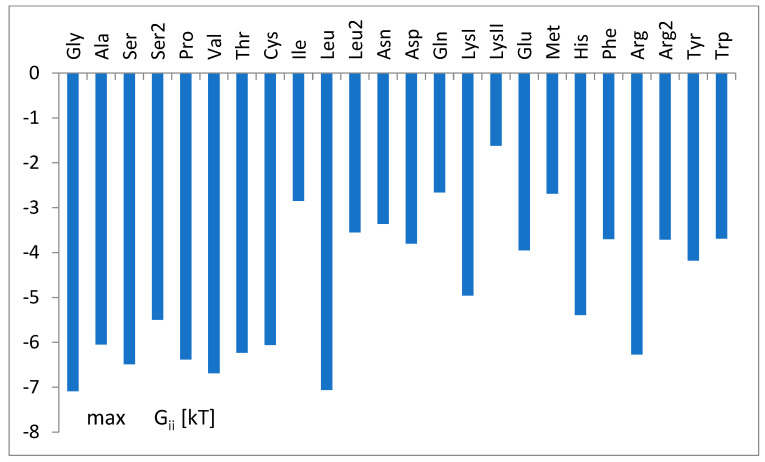
The energy of the less bound strands, maxΔG_ii_, for different classes. The tRNA^aa^ classes are presented according to increasing amino acid molecular weight.

The energy of the less bound strands, max ΔG_ii_, for different classes is shown in Figure 17. A decrease is shown in the binding strength with an increase in the molecular weight of transported amino acids in the range of Gly–LysII, then a slight increase. The weak free energy of binding is −1.62 k_B_T.

## 4. Discussion

Although universal genetic code is a major informatics factor governing the development and functioning of all organisms, there is no rational argument suggesting that it is the only form of natural code. For proper functioning, many life processes require other universal identifiers, enabling error-free recognition. A three-letter (positions) universal genetic code enough for translational reading may not be enough during charging tRNA with an appropriate amino acid via aminoacyl-tRNA synthetase. Preliminary numeric experiments indicate that, when we think about the assignment of the tRNA strand to a given amino acid class, the third position of the anticodon, position 34 of the tRNA strand (Figure 1), is not as important as a dominating anticodon tandem; namely, positions 36 and 35 (Figure 3). We propose to use the name “tandem” because nucleotides at these two positions usually occur together in the discussion of nucleotide importance. The 41st place out of 77, which position 34 takes in the correlation ranking (Table 2), entails practical meaning in machine learning. For example, the classifier performing the tRNA classification task of correctly choosing one of the two amino acids coded by the same anticodon tandem (e.g., Gln and His) will not prefer the nucleot(s)ide in position 34, as is the rule in the translation of the universal genetic code. It will recognize other, statistically more important components. Thus, if we assume that training of a classifier manifests the features of a natural enzymatic process of tRNA recognition and that the final single result of the classification corresponds to the enzymatic load of amino acid, it leads to the conclusion that, in the process of tRNA charging, the empty tRNA transporter may also effectively expose nucleot(s)ide(s) other than the anticodon to aminoacyl-tRNA synthetase. Marginalization of the third position of the anticodon during amino acid load, a position that is very important in the translation process, may be related to the specificity of the nucleotide–protein interactions (H-bonds, salt bridges, and hydrophobic effect) in the area of the anticodon site during t-RNA attachment to the synthetase. Regardless of the loose by “wobble” effect, complementary specify in position 34 has to be maintained for future translation in the ribosome; however, in some tRNA classes, it may not be guaranteed by the local interaction of the synthetase with a single nucleotide in this position. In such cases, other well-defined nucleotides, even outside the anticodon loop and stem, have to be applied. They may also speed up the recognition process. Thus, it is expected that so-called “identity nucleotides” are a small set of nucleotides determining the identity of tRNA; more precisely, carrying chemical groups that often interact with amino acids on the synthetases.

Another example of the importance of the identity nucleotides is the 6-fold degenerate amino acids (Leu, Ser, and Arg), and tRNAs may expose the two different leading nucleotide tandems in the anticodon. In this case, identity nucleotides may reduce the uncertainty level in positions 35 and 36 and prohibit charging errors, in effect also speeding up the binding process.

On the other end of the tRNA strand, the proper fit of the tRNA acceptor site to the catalytic center of synthetase may also require additional identifying nucleotides, which may be especially essential in recognizing the tRNA for the same code amino acid via the synthetases off different classes (LysI and LysII).

The strand positions less important than 34, e.g., 8, 20, 33, and 74–76 (Figure 3), may be related to the universal third-order structure of the tRNA strand and the acceptor site CCA-3′.

As nucleotide-specific interactions between aminoacyl-tRNA synthetases and their cognate tRNAs ensure accurate RNA recognition and prevent the binding of noncognate substrates, reducing further translational errors, the above examples highlight the importance of identity nucleotides.

Machine learning algorithms are computational models that allow computers to automatically improve their findings thanks to the experience gained while analyzing the training data sets. Their advantages are the fast processing of big data and objectivity, which is especially important in analyzing biological data. Machine learning classifiers are algorithms that automatically assign data points to classes. As such, they are great tools for modeling the processes of recognition decisions. A simple logistic classifier uses simple logistic regression to predict a binary variable (0, 1) assigned to a decision. This technique assumes that the relationship between the natural log of the odds ratio and the measurement variable described by the so-called predictor function is linear. In the discussed case, the predictor function quantitatively sums the presence of selected nucleotides, assigning them appropriate weights manifesting their importance.

The Weka SimpleLogistic classifier was verified in the preliminary numeric experiments as the best classifying algorithm in 10-fold cross-validation and 66% split tests and was finally chosen among others (Table 3). The useful feature of this classifier is the automatic explicit indication of the most important attributes (nucleotides) and their values (weights). The number of algorithmically selected nucleotides depends on the stopping criterion of LogitBoost iterations. The chosen option of minimizing the training misclassification error results in fewer setups of selected attributes than in the case of AIC or cross-validation options.

The final classification task with a full training set indicates the important positions in the tRNA strand in recognizing the proper class. Their usage (Figure 4a,b) correlates (cc = 0.63) with the ranks assigned by the CorrelationAttributeEval (data partially presented in Table 2). The most useful are the anticodon loop area (34–37) and positions 2, 21, 48, and 73, localized in the Acc-stem, D-loop, V-loop, and 3′ end regions. It is assumed that the revealed contents of these places represent the nucleotides, which are necessary for full identification. Thus, they were named identity positions and nucleotides.

The revealed identity positions and nucleotides are presented in Table 4. The shown class cases obey 2–8 places, filled with 14 different nucleot(s)ides, i.e., A, B, C, D, G, K, M, P, Q, U, 6, 8, and # (for symbol meanings, see Supplementary Materials, Table S1). They also contain the empty positions (-) and the exclusion rules (~) indicating unfavorable staffing. The presented attributes (positions) with their values (nucleotides) are theoretically the most representative group of features properly determining the values of the linear learners (predictors) for the correct determination of the predicted classes. In some cases, there were two or three different fillings proposed at position 34, which was only indicated in the eight classes.

The identity positions use the most representative components of the consensus strand in the class of tRNAs loaded by a given amino acid (Table 5). Only 13/129 (10%) positions do not meet consensus meaning. Global consensus, as in positions 74–76, was not useful for specific tRNA^aa^ recognition.

The identity nucleotides are not always fully arranged as a collective in a real single adaptor molecule, but they are at least partially visible in the strands of a given tRNA class (Figure 5). Full sets of predicted class identity nucleotides occurred at least in one strand and were observed in 67% (16/24) of tRNA classes (Figure 6). In other classes, the maximum occurrence exceeded 71% (5/7) of possible identity positions per strand (Glu).

Some ensembles of identity nucleotides, wider than the anticodon tandem, can serve as universal tRNA^aa^ class markers, i.e., they are entirely present in all strands of only one tRNA^aa^ class (Table 6). The above findings raise questions about the direction of the evolution of these sites and the possible informational role and importance of specially marked amino acid transporters.

Some ensembles of identity nucleotides, even without those from positions 35 and 36, exhibit high-class specificity. A total of 16 such ensembles in 76 issues within 511 strands were observed as unique, i.e., observed only in one class (Table 7), but not always. They contain 2–6 positions written with an 11-letter alphabet, which also obey empty positions or the attributes with the opposite impact. They may be a large fraction among identity nucleot(s)ides of a given class (Figure 5), but they may not be common in all strands. Such unique extra-anticodon ensembles may conserve the former predicted classification in the case of a modified anticodon tandem. This mechanism could function during the evolution of the genetic code, producing its extra degeneration. There may be some tracers of this phenomenon, e.g., position 21A in Arg and Arg2 and position 48~- in Leu and Leu2 (Table 4). This may also be the source of translation errors.

In the case of a classifier based on a machine learning algorithm correctly classifying representatives of classes occurring in nature, the probability of its decisions must be consistent with the probability of occurrence of real physicochemical processes naturally determining these classes. In analyzing the formation of tRNA classes, we assumed that these are the thermodynamic processes of overcoming the potential barrier during the fitting of the tRNA strand to the corresponding ligase. Thus, the central assumption of the proposed theoretical model of machine learning simulation of the tRNA binding to aminoacyl-tRNA synthetase is the correspondence between the biologically probable diversity of tRNA^aa^ strands revealed by the AI classifier and the thermodynamic probability. This allows for the mathematical alignment of the logarithm of odds for classification task and logits for thermodynamically driven tRNA and synthetase binding (Equation (8)), i.e., ln(odds’_i_) = logit(p_ii_), where odds’ is the measure of the success in the numeric experiment and p_ii_ is the probability of binding described by the Boltzmann distribution (Equation (4)). This allows for the expression of the change in the free energy of binding, ΔG_ii_, by the predictor function f_i_, which may be treated as dimensionless energy (Equation (9)). Thus, the predictor, including the presence of selected nucleotides, becomes a mathematical model of free energy change related to this nucleotide, namely, its interaction with aaRS.

The overall picture for all classes of the energetic input, ΔG_ijk_, of a given tRNA position to a total free energy change is presented in Figure 7a. It shows the energetically rescaled values (Equation (10)) of the coefficients (par_ijk_) of the linear predictor functions (Equation (1)) representing the relative effect of a given filling for the value of the predictor of a given class. The maximal value of a given predictor at a given nucleotide content leads to assigning a classified tRNA strand to a corresponding class. In Figure 7b, a detailed example is shown for a single predictor of tRNA^Gln^ class. It calculates five positions of a negative energy input (attraction) and three positions of a positive input (repulsion) if filled with indicated nucleotides. In this work, the positive values of energy input are interpreted as exclusion rules, e.g., ΔG_ijk_ > 0 for nucleotide “A” in position 44, which implicates the rule “44~A”, i.e., the presence of “A” in this position testifies against tRNA^Gln^ class; all other nucleot(s)ides are neutral.

**Figure 18:**
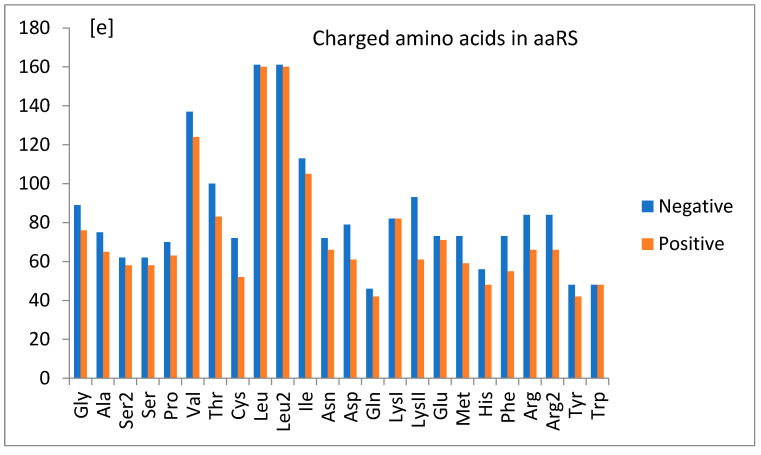
The electric charge [e], positive and negative, of aminoacyl-tRNA synthetases at a pH of 7.0 related to amino acids. The net charge is, in most cases, negative.

The nucleotide-independent part of free energy change, ΔG^0^_ii_ (Figure 8), calculated according to the bias of predictor function (Equation (1)) and the theoretical formula (Equation (11)), was always positive, which corresponds to the repulsion. This term represents part of the energy unexplained strictly by the nucleotidic attributes of the simple logistic model. On the other hand, a simple analysis showed its moderate correlation (cc = 0.46) with the common net negative charge of amino acids of aminoacyl-tRNA synthetases at a pH of 7.0 (Figure 18). As seen in Figure 9a,b, tRNA exposes its negatively charged sugar–phosphate backbone toward an overwhelmingly negative enzyme, which may result in electrostatic repulsion. This repulsion is reduced in the cases of tRNA with the crucial third anticodon nucleotides, which have to bind stronger to be properly recognized (see Figure 11). Thus, it is reasonable to assume that ΔG^0^_ii_ describes electrostatic repulsion between the tRNA backbone and the amino acids of synthetase.

The average change in the free energy of binding ΔG_ii_ was estimated at −8.6 to −4.0 k_B_T (Figure 10). A decreasing trend in the binding energy with the increase in the molecular mass of amino acid until LysII, then a slight increase, was observed. Its comparison (Figure 11) with the energy barrier for the attachment, ΔG^for^_ii_, approximated by ΔG^0^_ii_ (Equation (13)), led to the estimation of the strength of binding, i.e., the energy required to reverse the process, ΔG^rev^_ii_ = ΔG_ii_ − ΔG^for^_ii_. The strongest attraction was determined for the tRNA^Gly^ class, ΔG^rev^_ii_ = −15.2 [k_B_T]. The weakness attraction, ΔG^rev^_ii_ = −6.6 [k_B_T], was found for the tRNA^LysII^ class.

Some simultaneous variations in the free energy change with the increase in the number of identity positions, N_ip_ (Figure 12), were observed. Thus, the reversal energy limited only to the anticodon tandem, ΔG^rev^_ii_(tan), increases (the attraction of the tandem decreases), but the rest of the reversal energy, without the anticodon tandem, ΔG^rev^_ii_|tan, and part-forward of the energy, ΔG^for^_ii_, decrease (attraction increase). This counterplay of the outside-codon factors is not able to balance fully the tandem energy variation, so the total free change slightly increases (Figure 13) with the number of identity positions.

Specifically, an unambiguous interpretation of the sequence-independent energy component ΔG^0^_ii_ as the long-range term ΔG^for^_ii_ (Equation (13)) in the “crossing the energy barrier” model (Figure 2) may cause some issues. There could be other sequence-independent interactions that are short ranged (but do not contribute to the sequence-specific recognition process). As the value ΔG^for^_ii_, calculated as ΔG^for^_ii_ = ΔG^0^_ii_, decreases with the number of identity positions N_ip_ (Figure 12), this may suggest that for the appropriately large number of the identity nucleotides, the parameter b_i_ (Equation (11)) and the corresponding repulsion fall to zero independently of increasing the proximity of molecules. Thus, at first approximation, short-range repulsion can be neglected, and ΔG^for^_ii_ mainly reflects the long-range interaction energy.

The “strong” nucleotides (G and C), with a theoretically possible three-hydrogen bond in interactions with other RNA or protein in positions 35 and 36, bind aminoacyl-tRNA synthetases stronger than “weak” nucleotides (A, U) (Figure 14). This may suggest the important role of hydrogen bonding. The average data in the other positions for all analyzed classes were insufficient. The dependence of the total energy of the attraction on the actual number of possible hydrogen bonds, N_HB_, in different identity ensembles of the tRNA^Glu^ class (Figure 15) seems to confirm the above findings. At the constant anticodon tandem, the attraction of the other identity nucleotides increases with the actual number of hydrogen bonds, so the actual ΔG^rev^_ii’_ decreases.

The examples of recalculations for glutaminyl-tRNA synthetase (Figure 16a), histidyl-tRNA synthetase (Figure 16b), and lysyl-tRNA synthetase (Figure 16c) using consensus tRNA^aa^ strands suggest that for proper recognition, the change in binding free energy should decrease below a certain level, characteristic for a given class. The weaker bound or repelled tRNAs are not recognized. The energies of the real less bound strands, max ΔG_ii_, for different classes, are shown in Figure 17. They estimate the minimal binding energy levels.

The misacetylation as the source of mistranslation occurs approximately ten times less frequently than misreading [19]. It should also be investigated as a potential source of translational errors. The data in Figure 16c show that tRNA^Asn^ is the best candidate to be misrecognized by lysyl-tRNA synthetase of class II.

Generally, the energies presented in a holistic approach in Figure 16a–c and Figure 17 can be skewed and thus misleading due to overrepresentation or underrepresentation. To avoid this effect, the record duplicates were removed from a data set, and the unique strands representing a wide spectrum of possible tRNA occurrences in nature were considered (see Section 2.1). In the theoretical model (Equation (3)), it was assumed that the [Aminoacyl-AMP aaRS] complexes for all coded amino acids are equally available and the classes in the machine learning classifier are equally accessible. These conditions, being reminiscent of the issue in statistical mechanics regarding the principle of equal a priori probabilities, may avoid additional skewness. The entropic component is not a topic here, but at an assumed constant temperature, it does not influence the change in the Gibbs free energy.

**Figure 19:**
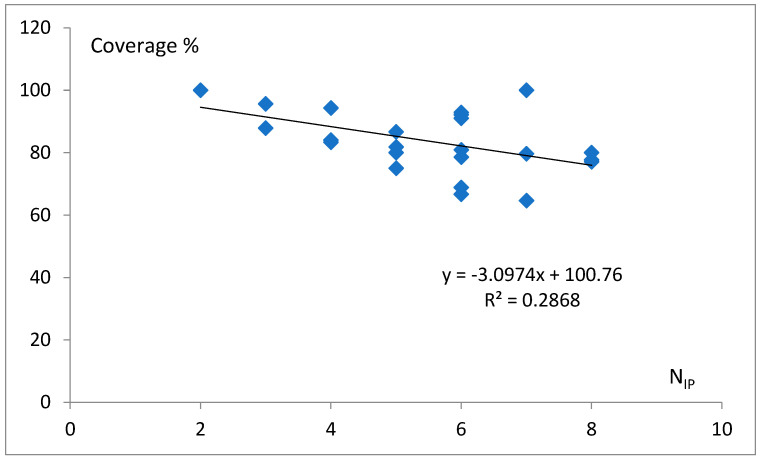
The coverage of identity nucleot(s)ides vs. the maximal number of identity points, N_IP_. Only non-repulsing identity nucleotides were calculated.

The coverage of identity nucleot(s)ides, i.e., the ratio of those that occurred to possibly occurred in a given class (please see the example in Figure 5), varies with the maximal number of identity positions, N_IP_ (Figure 19). It also decreases with the number of identity positions, which raises the question if it may be an evolutional trend. The coverage, similar to the abundance [20], might be a useful parameter in the biophysical modeling of biological processes.

**Figure 20:**
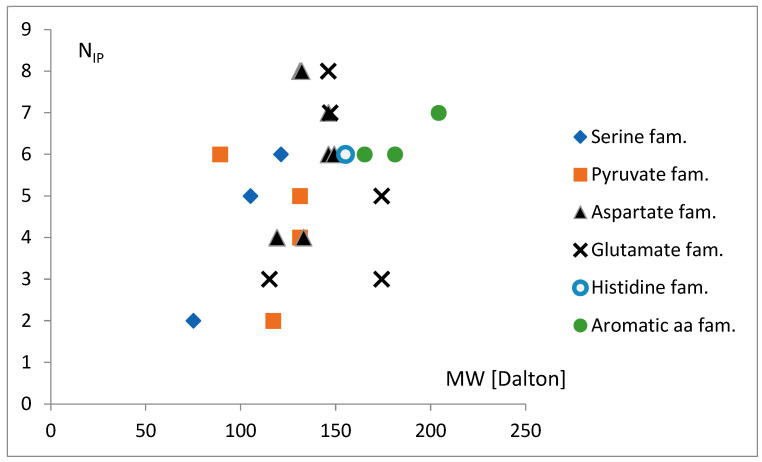
The molecular weight of charging amino acid, MW, and N_IP_ of the tRNA^aa^ classes shown in the field of discussed parameter values, with the indicated aa metabolic families, i.e., serine (Ser, Ser2, Gly, and Cys), pyruvate (Ala, Val, Leu, and Leu2), aspartate (Asp, Asn, LysI, LysII, Met, Thr, and Ile), glutamate (Glu, Gln, Pro, Arg, and Arg2), histidine (His), and aromatic (Phe, Trp, and Tyr).

When simultaneously analyzing the field of the two parameters possibly related to the evolutionary history of genetic code, i.e., the molecular weight of charging amino acid, MW, and N_IP_, a specific manner was found in which the tRNA^aa^ classes of aa belonging to the same metabolic families cover the area of discussed values (Figure 20).

According to this picture, the tRNA^aa^ for aa from the serine and pyruvate families is characterized by a lower range of MW and N_IP_ values. On the other hand, the histidine and the aromatic family contain MW and N_IP_ from the ranges of the higher values. The aspartate and glutamate families cover the range of moderate MW and the wide spectrum of N_IP_. One may conclude that the weak aa mass and the weakly identified tRNA, serine, and pyruvate families completely emerged at the beginning of the evolution of the code, much earlier than the final histidine and aromatic families. In this scheme, the other families were created throughout the entire period of the evolution.

**Figure 21:**
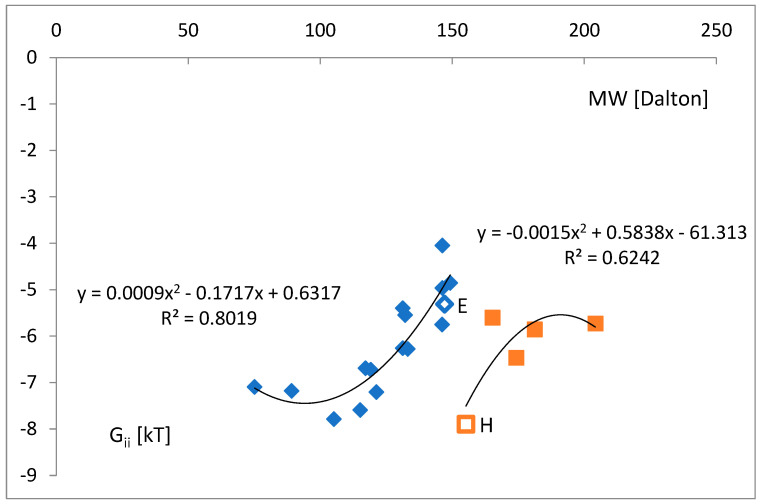
The average free energy change ΔG_ii_ vs. molecular weight of the corresponding amino acid. Two weight groups of amino acids were distinguished: below and above 150 [Dalton]. Empty markers indicate E-glutamic acid and H-histidine. Histidine has the most similar consensus strand to glutamic acid and vice versa.

Moreover, the average free energy change in a given tRNA^aa^ class increases with the molecular weight of the corresponding amino acid (Figure 21). Two weight groups of amino acids were distinguished: below and above 150 [Dalton]. The initial trend for Gly-Met (blue) results in a fragile binding above the mass of methionine, which might stop the aminoacylation of tRNA. It is likely that this trend was evolutionarily changed to the stronger binding trend His-Trp (red) due to small mutations in the previously used stronger bound tRNA strands, even amplifying its binding. The consensus strand (Table 5) of histidine (H) is the most similar to glutamic acid (E) and vice versa. The consensus strands are the closest in the entire analyzed set. The tRNA for histidine includes, as the only one, strongly attracts glycine at position −1 (Table 4, Figure 7a).

It is reasonable to expect that the tRNA of the amino acids with the later originated genetic code has more identity positions, the classes of earlier becoming code are better completed, and they transport lighter amino acids [21]. The dependence of the coverage on the number of identity positions (Figure 19) and the specific distribution of aa families on the parameters plane, MWxN_IP_ (Figure 20), seem to support these expectations. As a result, the idea to treat N_IP_ as a determinant of the evolutionary progress can be postulated, and only alone anticodons or very short ensembles of identity nucleotides may play a role at the beginning of the evolution, i.e., a two-letter code evolution. However, their informative role became too low and came to be supported by the bigger sets of spatially distributed elements. This is especially clearly seen in the case of the anticodons of such pairs as Asp-Glu, Arg2-Ser2, Ile-Met, and Cys-Trp, where the third position is essential for proper translation and should be correctly recognized during amino acid load. These pairs could evolve from the two-letter coded amino acids loaded onto the strains containing the two separate subsets of the extra-anticodon unique identity nucleot(s)ides, which, at some stage of evolution, became processed by the two different synthetases. Then, unique extra-anticodon positions could avoid docking errors at the third position of the anticodon site. This enabled evolutional differentiation of the third position of the anticodon and the charging amino acids at the first and the second fixed positions of the anticodon tandem. A similar mechanism, obeying entire sets of identity nucleot(s)ides, could also permit the differentiation of the Lysyl-tRNA-synthetases classes (LysI and LysII). There are probably many other consequences of the existence of the identity nucleot(s)ides and their unique subsets within tRNA strands, e.g., determining the third-order structure, which requires a detailed analysis in the future. This is in favor of the positive answer to the central research question of this paper regarding the importance of identity nucleotides beyond anticodon.

**Figure 22:**
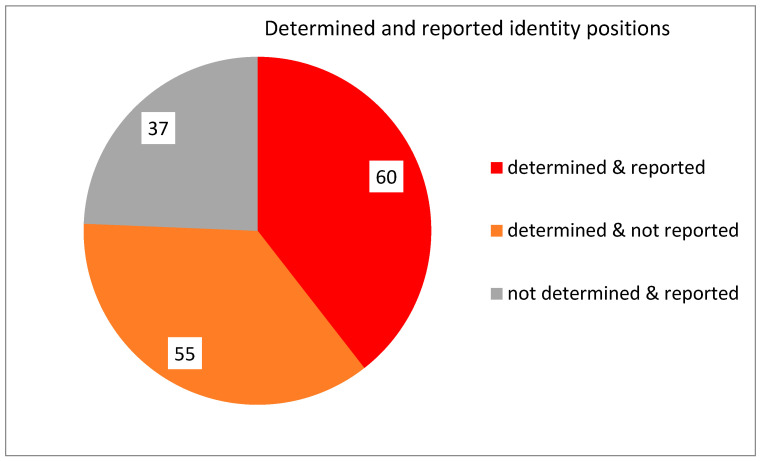
The identity positions determined by the model and reported in the literature. Note: to avoid overestimation, in the report’s analysis [22], the complementary nucleotide pairs were counted as units. In the analysis of the positions determined by the presented model, class LysI was omitted.

The identity positions determined by the presented model (Figure 22) cover 62% (60/97) of positions conserved in the three domains of life, as reported in a recent review [22]. The 55 predicted positions in all classes are not cited in this review, and the 37 reported positions are not predicted. This may be due to the limitations of the literature study and the performance of the trained model (which might not always be the highest) at assumed parameters. Both factors can be improved in future research.

The independent results of the Gibbs free energy of tRNA-aaRS interactions are not known to the authors. The only known report of the estimation of long-range electrostatic attractions presented in the work [23] reveals the order of a few k_B_T, which is similar in magnitude to that observed in the results of our models. In the authors’ opinion, the presented application of the machine learning tool and the thermodynamics approach leads to interesting results that are hard to obtain using other methods, and this is why they are worth publishing.

The discussed importance of identity nucleotides makes the presented findings, such as universal markers, unique ensembles, or Gibbs free energy of tRNA-aaRS binding, important to the broader field of molecular biology or tRNA research.

Despite the present paper focusing on the tRNA, some evolutional aspects, among others indicating the number of identity positions as an important parameter related to evolution, are a good basis for the future study of the coevolution of tRNA and aaRS. Although one may expect that due to transcriptional and translational mechanisms, the synthetases evolve slower than the corresponding tRNA strands and could not be observed, the observed divergence of tRNA-ligase classes (LysI and LysII) is the first gate to this area.

## 5. Summary

A total of 24 linear logistic regression models selecting identity nucleotides (or nucleosides) and quantitatively evaluating their importance, thus predicting different classes of t-RNA load with the corresponding amino acid, were developed using the machine learning classification method. The favorite location of identity nucleotides appears in the different parts of the tRNA strand, i.e., in the anticodon loop, especially in the tRNA anticodon and position 37; in the acceptor stem, at the 5′ end, positions 2; at the 3′ end, position 73; in D-loop, position 21, and also in the variable loop, position 48. They agree with the components of the consensus strand in a class of tRNAs loaded using a given amino acid. According to the proposed theoretical model of machine learning simulation with the accepted working hypothesis, the values of the free energy to enter the recognition state in the process of tRNA loading were obtained, and the inputs from the identity nucleotides and tRNA strand backbone were distinguished. Almost all predictions indicate leading anticodon tandems defining the first and the second position of the anticodon (positions 35 and 36 of tRNA strand) and the small sets (up to six positions) of the other nucleotides, with the natural identity nucleotides being the most influential in the free energy balance. The magnitude of their input to this energy depends on the position in the strand, favoring positions −1, 35, and 36. The role of position 34 is relatively smaller. The identity attributes may not always be fully arranged in a real single tRNA molecule but were comprehensively present in a given tRNA class. Some subsets of the identity nucleotides, together with the anticodon, may be treated as universal class markers. Some other subsets, considered even without anticodons, are only present in the tRNA of a given class, but not always. The analysis of the individual logistic models shows that the absolute value of the energy of binding the anticodon tandem, 35–36, decreases with an increasing number of identity positions and with a decreasing possible number of hydrogen bonds. In these conditions, the absolute value of the energy of binding of other identity nucleotides increases. All models indicate the nucleotide-independent energy of the repulsion tRNA backbone, decreasing with the number of identity nucleotides. It was also shown that the total free energy change in entering the recognition state increases with the amino acid mass, making this process less spontaneous. The identity nucleotides, apart from the anticodon, may add some additional binding energy to overcome a certain energy level specific to a given amino acid class. The stability of universal genetic code is supported by a precisely controlled quantity of binding energy in tRNA–ligase interaction. However, during evolution, this process was not efficient enough to keep an unchanged level of strong binding, such as for early tRNA transporting of low-mass amino acids. The strand coverage by the identity positions decreases with the number of identity positions. On the other hand, an increase in the molecular weight of carried amino acids and the diversity of corresponding metabolic families were observed. As the number of identity positions may indicate the evolutional progress of a given tRNA class, the results of this study may be useful in the future analysis of the evolution of tRNA and coevolution of tRNA and aaRS.

## 6. Conclusions

Identity nucleot(s)ides may be revealed using the machine learning (ML) classification method.

The evolutional trend of the tRNA sequences toward completing identity nucleotides may be postulated.

This phenomenon, as a consequence, may finally lead to the emergence of specific nucleotidic markers of class and unique subsets of the nucleotides appropriately scattered outside the anticodon site, which may guarantee more precise control of the interaction of tRNA and aminoacyl-tRNA synthetase during the process of the amino acid load, and thus less erroneous protein synthesis.

A certain level of change in free energy of binding is required for aminoacylation of tRNA.

Identity nucleotides help to maintain the required free energy level for different anticodon contents.

## Figures and Tables

**Table 1:** A list of analyzed tRNA amino acid classes, tRNA^aa^.

tRNA^aa^ Class			
aa	Charging Amino Acid	Positions 35 and 36	Remarks
Ala	Alanine	G C	2-letter gen. code
Arg	Arginine	C G	degenerate pos. 36
Arg2	Arginine	C U	degenerate pos. 36
Asn	Asparagine	U U	3-letter gen. code
Asp	Aspartic acid	U C	3-letter gen. code
Cys	Cysteine	C A	3-letter gen. code
Gln	Glutamine	U G	3-letter gen. code
Glu	Glutamic acid	U C	3-letter gen. code
Gly	Glycine	C C	2-letter gen. code
His	Histidine	U G	3-letter gen. code
Ile	Isoleucine	A U	3-letter gen. code
Leu	Leucine	A G	degenerate pos. 36
Leu 2	Leucine	A A	degenerate pos. 36
LysI	Lysine	U U	synthetase class I
LysII	Lysine	U U	synthetase class II
Met	Methionine	A U	3-letter gen. code
Phe	Phenylalanine	A A	3-letter gen. code
Pro	Proline	G G	2-letter gen. code
Ser	Serine	G A	deg. pos. 35 36
Ser2	Serine	C U	deg. pos. 35 36
Thr	Threonine	G U	2-letter gen. code
Trp	Tryptophan	C A	3-letter gen. code
Tyr	Tyrosine	U A	3-letter gen. code
Val	Valine	A C	2-letter gen. code

**Table 2:** The values of the correlation rank for the most important attributes of those presented in Figure 3.

Rank Position	tRNA Sequence Position	Correlation Rank Value
1	35	0.1942
2	36	0.1925
3	46	0.1659
4	47	0.162
5	48	0.1612
6	73	0.1375
7	11	0.1263
8	24	0.1213
9	45	0.1172
10	13	0.1162
…	…	…
41	34	0.0752

**Table 3:** Predictions of the trained candidate classifiers.

Classifier	10 Fold Cross-Validation	66% Split	Full Training Set
SimpleLogistic	94.5205	92.5287	100
LibLINEAR	93.9335	85.6322	100
RandomForest	93.9335	83.908	100
SMO	92.3679	81.0345	100
Dl4jMlpClassifier	86.6928	78.1609	100
ZeroR	8.0235	8.046	8.0235

**Table 4:** The identity nucleot(s)ides for different tRNA^aa^ classes determined using the SimpleLogistic classifier. Symbol definitions are in Supplementary Materials, Table S1.

Ala	3G	17D	35G	36C	70U	71C		
Arg	21A	35C	36G					
Arg2	21A	35C	36U	69G	72U			
Asn	5C	14U	35U	37 6	39C	41G	63~C	73~A
Asp	34G8Q	35U	36C	73G				
Cys	9G	34G	35C	36A	63G	73U		
Gln	1~G	35U	36G	41C	44~A	58~A	70A	71C
Glu	12C	16A	24A	35U	36C	39C	50A	
Gly	35C	36C						
His	−1G	2C	17U	35U	36G	37G		
Ile	2G	7~G	35A	36U	37 6	40G	41G	70~G
Leu	35A	36G	48~-	55G				
Leu2	12A	13~C	35A	36A	48~-			
LysI	4C	18~-	28C	35U	36U	43~G	71C	
LysII	7~G	29U	34)~G	35U	36U	67~G		
Met	7G	11G	31P	34MCB	35A	36U		
Phe	21G	23A	34G#	35A	36A	44G		
Pro	35G	36G	37K					
Ser	25A	35G	36A	48~-	73G			
Ser2	20-	34 7G	35C	36U	46~-			
Thr	2C	35G	36U	73U				
Trp	22G	34B	35C	36A	70C	72U	73~U	
Tyr	21C	34G	35U	36A	45-	63~C		
Val	35A	36C						

**Table 5:** The consensus representatives of TRNA^aa^ strands are consistent, and inconsistent, with predictions of the SimpleLogistic model. Symbol definitions are in Supplementary Materials, Table S1.

	Ala	Arg	Arg2	Asn	Asp	Cys	Gln	Glu	Gly	His	Ile	Leu	Leu2	Lys1b	Lys2b	Met	Phe	Pro	Ser	Ser2	Thr	Trp	Tyr	Val
−1	-	-	-	-	-	-	-	-	-	G	-	-	-	-	-	-	-	-	-	-	-	-	-	-
1	G	G	G	G	G	G	G	G	G	G	G	G	G	G	G	G	G	C	G	G	G	A	G	G
2	G	G	C	U	A	G	G	C	C	C	G	C	U	G	A	C	C	G	G	A	C	G	G	G
3	G	G	C	C	C	C	U	C	G	C	G	U	C	G	C	C	C	G	A	C	C	G	A	U
4	G	C	C	U	A	U	G	C	G	G	C	A	A	C	U	U	G	C	G	A	G	G	G	U
5	G	C	C	C	C	A	C	U	G	U	C	G	G	C	G	G	A	G	G	A	A	G	G	C
6	C	C	C	U	G	C	C	C	G	U	U	U	G	C	G	C	G	A	C	A	U	C	G	C
7	A	G	G	G	G	A	A	G	G	A	U	A	A	G	U	G	A	G	G	G	U	G	G	G
8	U	U	U	U	U	U	U	U	U	U	U	U	U	U	U	U	U	U	U	U	U	U	U	U
9	A	A	A	K	A	G	K	G	A	A	A	G	G	A	A	A	A	A	G	G	A	A	A	A
10	G	G	G	L	G	G	G	G	G	G	G	L	G	G	L	G	L	G	G	G	G	G	G	G
11	C	C	C	C	U	C	U	U	U	U	C	C	C	C	C	C	C	C	C	C	C	U	C	U
12	U	U	U	G	A	C	G	C	U	U	U	M	M	U	U	U	U	C	M	C	U	U	G	U
13	C	C	U	C	P	G	P	P	U	P	C	G	G	C	C	C	C	C	G	-	C	C	A	P
14	A	A	A	A	A	A	A	A	A	A	A	A	A	A	A	A	A	A	A	A	A	A	A	A
15	G	A	A	A	G	G	G	G	A	G	G	G	G	G	G	G	G	G	G	G	G	A	G	G
16	C	D	U	D	U	C	D	A	U	D	U	D	D	C	D	D	D	C	D	-	D	U	U	D
17	D	-	-	C	-	-	-	-	-	-	D	-	-	C	C	D	D	U	-	-	-	-	-	-
18	-	-	-	-	-	-	-	-	-	-	-	-	-	A	-	-	-	U	-	-	-	-	-	-
19	G	G	G	G	G	G	G	G	G	G	G	G	G	G	G	G	G	G	#	G	G	G	G	G
20	G	G	G	G	G	G	G	G	G	G	G	G	G	G	G	G	G	G	G	G	G	G	G	G
21	G	A	A	D	D	D	D	C	D	D	D	D	D	C	D	D	G	D	D	D	D	D	C	D
22	G	A	G	G	G	A	G	G	G	G	G	A	A	G	G	G	G	G	A	A	G	G	A	U
23	A	A	A	C	U	G	C	G	A	A	A	G	G	A	A	A	A	G	G	G	A	A	C	A
24	G	G	G	G	A	G	A	A	A	A	G	G	G	G	G	G	G	G	G	G	G	A	G	A
25	C	U	C	C	U	C	C	C	C	C	C	C	C	C	C	C	C	U	C	C	C	C	C	C
26	R	R	A	R	A	A	U	A	A	A	R	R	R	G	A	A	R	A	R	R	A	A	G	A
27	C	C	C	P	C	P	C	C	C	C	P	C	C	G	P	P	P	C	A	A	C	C	G	P
28	U	U	C	P	C	C	C	C	C	A	G	U	C	C	P	C	P	U	C	A	C	C	C	C
29	U	U	G	C	C	G	G	G	A	G	C	G	A	G	U	G	A	U	A	G	U	G	A	U
30	G	G	G	G	G	G	G	C	G	C	C	G	G	G	G	G	G	G	G	A	C	G	G	G
31	C	A	C	G	C	A	A	G	C	G	G	A	A	G	A	P	A	C	A	A	C	A	A	C
32	U	C	C	C	C	C	B	C	C	P	C	P	C	C	C	C	B	U	hc	C	C	C	C	U
33	U	U	U	U	U	U	U	U	U	U	U	U	U	U	U	U	U	U	U	U	U	U	U	U
34	I	I	{	Q	G	G	N	C	G	G	G	U	.	U	C	C	#	U	I	G	G	B	G	I
35	G	C	C	U	U	C	U	U	C	U	A	A	A	U	U	A	A	G	G	C	G	C	U	A
36	C	G	U	U	C	A	G	C	C	G	U	G	A	U	U	U	A	G	A	U	U	A	A	C
37	A	K	6	6	A	K	A	A	A	K	6	K	K	A	6	6	*	K	*	6	6	*	*	A
38	C	A	A	A	A	A	A	C	G	C	A	C	A	A	A	A	A	P	A	A	A	A	A	?
39	G	P	G	C	G	P	P	C	G	C	C	P	P	C	P	P	P	G	P	G	P	P	P	G
40	C	C	C	C	G	C	C	G	C	G	G	C	C	C	C	C	?	C	C	U	G	C	C	C
41	A	A	C	G	G	C	C	C	U	C	G	C	U	C	A	C	U	A	U	C	A	C	C	A
42	A	A	G	A	G	G	A	G	G	U	A	A	G	G	A	G	A	A	G	A	G	G	G	G
43	G	G	G	A	G	U	G	G	G	G	G	G	G	C	A	A	U	G	U	U	G	A	C	A
44	A	A	A	A	A	A	C	A	A	A	A	U	J	G	G	A	A	G	J	J	A	A	U	A
45	G	G	G	G	G	A	G	G	G	A	G	-	-	G	G	G	G	G	-	-	G	G	-	G
46	-	-	-	-	-	-	-	-	-	-	-	C	G	-	-	-	-	-	G	G	-	-	G	-
47	-	-	-	-	-	-	-	-	-	-	-	C	C	-	-	-	-	-	G	G	-	-	C	-
48	-	-	-	-	-	-	-	-	-	-	-	C	C	-	-	-	-	-	G	-	-	-	C	-
49	?	?	G	G	A	A	?	?	?	?	A	?	G	C	A	A	?	?	G	G	G	G	G	?
50	C	G	C	G	C	C	?	?	U	U	C	U	U	C	A	C	C	C	C	C	U	U	G	C
51	G	G	A	U	G	C	G	G	G	G	U	G	G	G	G	A	U	A	A	G	A	G	A	C
52	G	G	G	G	G	G	G	G	G	G	G	G	G	G	G	A	G	G	G	G	G	G	G	G
53	G	G	G	G	G	G	G	G	G	G	G	G	G	G	G	G	G	G	G	G	G	G	G	G
54	T	T	T	.	T	U	U	T	T	U	T	T	T	U	T	T	T	T	T	T	T	T	T	T
55	P	P	P	P	P	P	P	P	P	P	P	P	P	U	P	P	P	P	P	P	P	P	P	P
56	C	C	C	C	C	C	C	C	C	C	C	C	C	C	C	C	C	C	C	C	C	C	C	C
57	G	G	G	G	A	G	G	G	G	G	A	A	G	A	G	G	G	A	G	G	G	G	G	G
58	A	A	A	“	A	A	A	A	A	A	A	A	“	A	A	A	“	A	A	A	A	A	A	A
59	U	A	A	G	U	A	A	A	U	A	G	A	A	A	G	A	U	A	A	A	U	A	A	A
60	U	U	U	C	U	U	U	U	U	U	U	U	U	U	U	U	?	U	U	U	U	U	U	U
61	C	C	C	C	C	C	C	C	C	C	C	C	C	C	C	C	C	C	C	C	C	C	C	C
62	C	C	C	C	C	C	C	C	C	C	C	C	C	C	C	U	C	C	C	C	C	C	C	C
63	C	C	U	A	C	G	C	C	C	C	A	C	C	C	C	C	G	U	U	C	U	U	G	G
64	G	U	G	C	G	G	G	G	G	A	C	A	A	G	U	U	G	G	G	G	A	G	U	G
65	G	G	G	C	U	U	G	G	G	G	U	C	C	G	C	U	G	G	C	G	C	C	C	G
66	C	C	C	C	C	U	U	U	C	U	A	U	U	C	A	A	U	C	C	C	A	C	C	C
67	G	G	G	A	C	G	G	G	C	A	A	G	U	G	U	G	C	U	G	U	G	G	C	G
68	G	G	G	G	G	U	G	G	C	A	G	C	C	G	C	C	U	G	C	U	U	C	C	G
69	C	G	G	G	U	G	G	G	C	C	G	U	U	G	A	C	C	G	C	U	C	C	C	A
70	U	C	G	G	G	G	A	G	C	G	C	G	G	C	G	G	G	C	U	G	G	C	U	A
71	C	U	G	A	U	C	C	G	G	G	C	G	A	C	U	G	G	C	C	U	G	C	C	C
72	C	C	U	C	C	C	C	A	C	?	C	C	C	C	C	C	C	G	C	C	C	U	C	C
73	A	G	A	G	G	U	U	A	A	A	A	A	A	G	A	A	A	A	G	G	A	G	A	A
74	C	C	C	C	C	C	C	C	C	C	C	C	C	C	C	C	C	C	C	C	C	C	C	C
75	C	C	C	C	C	C	C	C	C	C	C	C	C	C	C	C	C	C	C	C	C	C	C	C
76	A	A	A	A	A	A	A	A	A	A	A	A	A	A	A	A	A	A	A	A	A	A	A	A

**Table 6:** The universal tRNA^aa^ class markers.

Ala	35G	36C	71C		
Cys	34G	35C	36A	73U	
Glu	24A	35U	36C		
LysI	4C	28C	35U	36U	71C

**Table 7:** Unique ensembles of identity nucleotides without those from positions 35 and 36. Symbol definitions are in Supplementary Materials, Table S1.

Ala	3G	17D	70U	71C		
Arg2	21A	69G	72U			
Asn	5C	39C	41G	63~C	73~A	
Asp	34 8	73G				
Cys	9G	34G	63G	73U		
Glu	12C	16A	24A			
His	−1G	2C	17U			
	2C	17U	37G			
Ile	2G	7~G	37 6	40G	41G	70~G
Met	7G	31P	34C			
	7G	31P	34B			
Phe	21G	23A	34#			
	23A	34#	44G			
Ser	25A	48~-	73G			
Ser2	20-	34G	46~-			
Trp	22G	34B	70C	72U	73~U	

## Data Availability

All data generated or analyzed during this study are included in this article and its Supplementary Material files. Further inquiries can be directed to the corresponding author.

## Supplementary Materials

The following supporting information can be downloaded at: https://www.mdpi.com/article/10.3390/life14101328/s1, Table S1: Nucleot(s)ides, their symbols, and one-letter codes; Table S2: Parameters of the model.
