# Supplementary material for: Determining the Identity Nucleotides and the Energy of Binding of tRNAs to Their Aminoacyl-tRNA Synthetases Using a Simple Logistic Model
Source: Life (Basel). 2024 Oct 18;14(10):1328. doi: 10.3390/life14101328 (PMC11509504; doi:10.3390/life14101328)
Supplement: Supplementary file 1 [file life-14-01328-s001.zip › life-3229346-supplementary.pdf]

Table S1. Nucleot(s)ides, their symbols, and one-letter codes

Nucleotide derivatives	Name	Symbol	One letter code
Adenosines	adenosine	A	A
	unknown modified adenosine	?A	H
	1-methyladenosine	m1A	"
	2-methyladenosine	m2A	/
	N6-isopentenyladenosine	i6A	+
	2-methylthio-N6-isopentenyladenosine	ms2i6A	*
	N6-methyladenosine	m6A	=
	N6-threonylcarbamoyladenosine	t6A	6
	N6-methyl-N6-threonylcarbamoyladenosine	m6t6A	E
	2-methylthio-N6-threonylcarbamoyladenosine	ms2t6A	[
	2'-O-methyladenosine	Am	:
	2'-O-ribosyladenosine (phosphat)	Ar(p)	^
	N6-(cis-hydroxyisopentenyl)adenosine	io6A	`
Cytidines	cytidine	C	C
	unknown modified cytidine	?C	<
	2-thiocytidine	s2C	%
	2'-O-methylcytidine	Cm	B
	N4-acetylcytidine	ac4C	M
	5-methylcytidine	m5C	?
	3-methylcytidine	m3C	'
	Lysidine	k2C	}
	5-formylcytidin	f5C	>
	2-O-methyl-5-formylcytidin	f5Cm	°
Guanosines	Guanosine	G	G
	unknown modified guanosine	?G	;

	1-methylguanosine	m1G	K
	N2-methylguanosine	m2G	L
	2'-O-methylguanosine	Gm	#
	N2,N2-dimethylguanosine	m22G	R
	N2,N2,2'-O-trimethylguanosine	m22Gm	|
	7-methylguanosine	m7G	7
	Archaeosine	fa7d7G	(
	queuosine	Q	Q
	mannosyl-queuosine	manQ	8
	galactosyl-queuosine	galq	9
Uridines	Uridine	U	U
	unknown modified uridine	?U	N
	5-methylaminomethyluridine	mnm5U	{
	2-thiouridine	s2U	2
	2'-O-methyluridine	Um	J
	4-thiouridine	s4U	4
	5-carbamoylmethyluridine	ncm5U	&
	5-methoxycarbonylmethyluridine	mcm5U	1
	5-methylaminomethyl-2-thiouridine	mnm5s2U	S
	5-methoxycarbonylmethyl-2-thiouridine	mcm5s2U	3
	5-methoxyuridine	cmo5U	V
	5-carboxymethylaminomethyluridine	cmn5U	!
	5-carboxymethylaminomethyl-2-thiouridine	cmnm5s2U	$
	3-(3-amino-3-carboxypropyl)uridine	acp3U	X
	5-(carboxyhydroxymethyl)uridinemethyl ester	mchm5U	,
	5-carboxymethylaminomethyl-2'-O-methyluridine	cmnm5Um	)
	5-carbamoylmethyl-2'-O-methyluridine	ncm5Um	~
	dihydrouridine	D	D
	pseudouridine	psi	P

	1-methylpseudouridine	m1psi	]
	2'-O-methylpseudouridine	psi m	Z
	5-methyl-2-thiouridine	m5s2U	F
	2'-O-dimethyluridine	m5Um	\
Thymines	thymine	T	T
	ribosylthymine	m5U	T
Inosines	inosine	I	I
	1-methylinosine	m1I	O
Wybutosines	wybutosine	yW	Y
	peroxywybutosine	o2yW	W
Other	empty position		-
	insertion		_
	unknown nucleotide		.

Table S2. Parameters of the Simple Logistic model

Class	Predictor function f_i
Ala	-7.21 +<br>[3=G] * 1.41 +<br>[17=D] * 1.32 +<br>[35=G] * 5.99 +<br>[36=C] * 3.13 +<br>[70=U] * 1.78 +<br>[71=C] * 1.41
Arg	-7.59 +<br>[21=A] * 1.64 +<br>[35=C] * 6.66 +<br>[36=G] * 7.2
Asn	-2.79 +<br>[5=C] * 1.67 +<br>[14=U] * 3.15 +<br>[35=U] * 2.3 +<br>[37=6] * 2.18 +<br>[39=C] * 1.41 +<br>[41=G] * 2.09 +<br>[63=C] * -1.74 +<br>[73=A] * -1.66
Asp	-5.07 +

	[34=8] * 3.13 +<br>[34=G] * 3.47 +<br>[34=Q] * 2.83 +<br>[35=U] * 2.48 +<br>[36=C] * 3.91 +<br>[73=G] * 1.05
Cys	-7.46 +<br>[9=G] * 1.49 +<br>[34=G] * 3.87 +<br>[35=C] * 2.63 +<br>[36=A] * 1.49 +<br>[63=G] * 1.83 +<br>[73=U] * 4.04
Gln	-1.44 +<br>[1=G] * -1.41 +<br>[35=U] * 2.53 +<br>[36=G] * 2.69 +<br>[41=C] * 1.57 +<br>[44=A] * -1.2 +<br>[58=A] * -1.62 +<br>[70=A] * 1.62 +<br>[71=C] * 1.74
Glu	-5.83 +<br>[12=C] * 2.41 +<br>[16=A] * 1.79 +<br>[24=A] * 1.6 +<br>[35=U] * 2.44 +<br>[36=C] * 4 +<br>[39=C] * 1.74 +<br>[50=A] * 2.17
Gly	-8.11 +<br>[35=C] * 7.22 +<br>[36=C] * 7.98
His	-5.43 +<br>[-1=G] * 8.34 +<br>[2=C] * 1.48 +<br>[17=U] * 2.41 +<br>[35=U] * 1.67 +<br>[36=G] * 2.3 +<br>[37=G] * 2.96
Ile	-3.44 +<br>[2=G] * 1.3 +<br>[7=G] * -2.05 +<br>[35=A] * 1.92 +<br>[36=U] * 2.61 +<br>[37=6] * 1.3 +<br>[40=G] * 1.89 +<br>[41=G] * 2.01 +<br>[70=G] * -1.39

<table>
<tr><td>Leu</td><td>-3.88 +
[35=A] * 3.27 +
[36=G] * 7.78 +
[48=-] * -3.25 +
[55=G] * 3.14</td></tr>
<tr><td>Lys1b</td><td>-3.37 +
[4=C] * 1.32 +
[18=-] * -3.46 +
[28=C] * 1.38 +
[35=U] * 2.35 +
[36=U] * 1.59 +
[43=G] * -1.4 +
[71=C] * 1.69</td></tr>
<tr><td>Lys2b</td><td>-2.58 +
[7=G] * -1.6 +
[29=U] * 1.13 +
[34=)] * 2.2 +
[34=G] * -1.43 +
[35=U] * 2.39 +
[36=U] * 3.41 +
[67=G] * -1.51</td></tr>
<tr><td>Met</td><td>-4.21 +
[7=G] * 1.64 +
[11=G] * 2.19 +
[31=P] * 2.7 +
[34=B] * 1.59 +
[34=C] * 2.3 +
[34=M] * 2.85 +
[35=A] * 2.08 +
[36=U] * 2.52</td></tr>
<tr><td>Phe</td><td>-6.4 +
[21=G] * 2.89 +
[23=A] * 2.29 +
[34=#] * 1.37 +
[34=G] * 1.55 +
[35=A] * 1.97 +
[36=A] * 4.29 +
[44=G] * 1.27</td></tr>
<tr><td>Pro</td><td>-7.72 +
[35=G] * 7.61 +
[36=G] * 6.49 +
[37=K] * 1.39</td></tr>
<tr><td>Ser</td><td>-5.25 +
[25=A] * 2.2 +
[35=G] * 6.72 +
[36=A] * 3.5 +
[48=-] * -1.97 +
[73=G] * 3.49</td></tr>
<tr><td>Thr</td><td>-7.36 +
[2=C] * 1.45 +
[35=G] * 7.57 +</td></tr>
</table>

	[36=U] * 4.59 +<br>[73=U] * 1.43
Trp	-4.87 +<br>[22=G] * 1.52 +<br>[34=B] * 1.41 +<br>[35=C] * 4.02 +<br>[36=A] * 2.78 +<br>[70=C] * 1.2 +<br>[72=U] * 1.76 +<br>[73=U] * -1.48
Tyr	-5.65 +<br>[21=C] * 1.66 +<br>[34=G] * 1.88 +<br>[35=U] * 4.12 +<br>[36=A] * 3.83 +<br>[45=-] * 2.42 +<br>[63=C] * -1.66
Val	-8.13 +<br>[35=A] * 7.22 +<br>[36=C] * 7.6
Arg2	-6.9 +<br>[21=A] * 2.81 +<br>[35=C] * 4.64 +<br>[36=U] * 3.81 +<br>[69=G] * 1.44 +<br>[72=U] * 2.16
Leu2	-1.69 +<br>[12=A] * 2.15 +<br>[13=C] * -1.35 +<br>[35=A] * 3.81 +<br>[36=A] * 3.4 +<br>[48=-] * -4.12
Ser2	-1.89 +<br>[20=-] * 1.69 +<br>[34=7] * 3.3 +<br>[34=G] * 1.57 +<br>[35=C] * 3.9 +<br>[36=U] * 2.85 +<br>[46=-] * -4.35
